# Three-dimensional structural dynamics and fluctuations of DNA-nanogold conjugates by individual-particle electron tomography

**DOI:** 10.1038/ncomms11083

**Published:** 2016-03-30

**Authors:** Lei Zhang, Dongsheng Lei, Jessica M. Smith, Meng Zhang, Huimin Tong, Xing Zhang, Zhuoyang Lu, Jiankang Liu, A. Paul Alivisatos, Gang Ren

**Affiliations:** 1 The Molecular Foundry, Lawrence Berkeley National Laboratory, Berkeley, California 94720, USA; 2 Department of Applied Physics, School of Science, Xi'an Jiaotong University, Xi'an 710049, China; 3 Materials Sciences Division, Lawrence Berkeley National Laboratory, Berkeley, California 94720, USA; 4 Department of Chemistry, University of California, Berkeley, California 94720, USA; 5 Department of Materials Science, University of California, Berkeley, California 94720, USA; 6 Center for Mitochondrial Biology and Medicine, The Key Laboratory of Biomedical Information Engineering of the Ministry of Education, Xi'an Jiaotong University, Xi'an 710049, China; 7 School of Life Science and Technology, Xi'an Jiaotong University, Xi'an 710049, China; 8 Frontier Institute of Science and Technology, Xi'an Jiaotong University, Xi'an 710049, China; 9 Kavli Energy NanoScience Institute, University of California, Berkeley, California 94720, USA

## Abstract

DNA base pairing has been used for many years to direct the arrangement of inorganic nanocrystals into small groupings and arrays with tailored optical and electrical properties. The control of DNA-mediated assembly depends crucially on a better understanding of three-dimensional structure of DNA-nanocrystal-hybridized building blocks. Existing techniques do not allow for structural determination of these flexible and heterogeneous samples. Here we report cryo-electron microscopy and negative-staining electron tomography approaches to image, and three-dimensionally reconstruct a single DNA-nanogold conjugate, an 84-bp double-stranded DNA with two 5-nm nanogold particles for potential substrates in plasmon-coupling experiments. By individual-particle electron tomography reconstruction, we obtain 14 density maps at ∼2-nm resolution. Using these maps as constraints, we derive 14 conformations of dsDNA by molecular dynamics simulations. The conformational variation is consistent with that from liquid solution, suggesting that individual-particle electron tomography could be an expected approach to study DNA-assembling and flexible protein structure and dynamics.

## Abstract (web summary)

The control of DNA-mediated assembly depends on a precise understanding of the three-dimensional structure of DNA-nanocrystal-hybridized building blocks. Here, the authors use cryo-electron microscopy and negative-staining techniques to investigate the morphology of DNA-nanogold conjugates.

## Graphical abstract

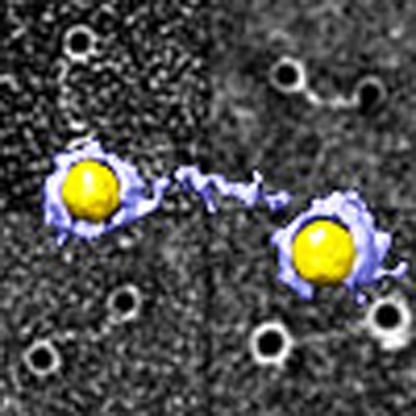

Organic–inorganic-hybridized nanocrystals are a valuable class of new materials that are suitable for addressing many emerging challenges in biological and material sciences^1^,^2^. Nanogold and quantum dot conjugates have been used extensively as biomolecular markers^3^,^4^, whereas DNA base pairing has directed the self-assembly of discrete groupings and arrays of organic and inorganic nanocrystals in the formation of a network solid for electronic devices and memory components^5^. Discretely hybridized gold nanoparticles conjugated to DNA were developed as a molecular ruler to detect sub-nanometre distance changes via plasmon-coupling-mediated variations in dark-field light scattering^3^,^6^. For many of these applications, it is desirable to obtain nanocrystals functionalized with discrete numbers of DNA strands^7^,^8^. In all of these circumstances, the soft components can fluctuate, and the range of these structural deviations have not previously been determined with a degree of rigour that could help influence the future design and use of these assemblies.

Conformational flexibility and dynamics of the DNA-nanogold conjugates limit the structural determination by X-ray crystallography, nuclear magnetic resonance spectroscopy and single-particle cryo-electron microscopic (cryo-EM) reconstruction because they do not crystallize, are not sufficiently small for nuclear magnetic resonance studies and cannot be classified into a limited number of classes for single-particle EM reconstruction. In addition, three-dimensional (3D) structure averaged from tens of thousands of different macromolecular particles obtained without prior knowledge of the macromolecular structural flexibility could result in an absence of flexible domains upon using the single-particle reconstruction method, for example, two ankyrin repeated regions of TRPV1 were absent in its atomic resolution 3D density map^9^.

A fundamental experimental solution to reveal the structure of a flexible macromolecule should be based on the determination of each individual macromolecule's structure^10^. Electron tomography (ET) provides high-resolution images of a single object from a series of tilted viewing angles^11^. ET has been applied to reveal the 3D structure of a cell section and an individual bacterium at nanometre-scale resolution^12^. However, reconstruction from an individual macromolecule at an intermediate resolution (1–3 nm) remains challenging due to small molecular weight and low image contrast. Although, the first 3D map of an individual macromolecule, a fatty acid synthetase molecule, was reconstructed from negative-staining (NS) ET by Hoppe *et al.*^13^, serious doubts have been raised regarding the validity of this structure^14^, as this molecule received a radiation dose hundreds of times greater than the reported damage threshold^15^. Recently, we investigated the possibility based on simulated and real experimental NS and cryo-ET images^10^. We showed that a single-protein 3D structure at an intermediate resolution (1–3 nm) is potentially achieved using our proposed individual-particle ET (IPET) method^10^,^16^,^17^,^18^. IPET, an iterative refinement process using automatically generated dynamic filters and soft masks, requires no pre-given initial model, class averaging or lattice, but can tolerate small tilt errors and large-scale image distortion via decreasing the reconstruction image size to reduce the negative effects on 3D reconstruction. IPET allows us to obtain a ‘snapshot' single-molecule 3D structure of flexible proteins at an intermediate resolution, and can be even used to reveal the macromolecular dynamics and fluctuation^17^.

Here we use IPET, cryo-EM and our previously reported optimized NS (OpNS)^19^,^20^ techniques to investigate the morphology and 3D structure of hybridized DNA-nanogold conjugates. These conjugates were self-assembled from a mixture of two monoconjugates, each consisting of 84-bp single-stranded DNA and a 5-nm nanogold particle. The dimers were separated by anion-exchange high-performance liquid chromatography (HPLC) and agarose gel electrophoresis as potential substrates in plasmon-coupling experiments. By OpNS-ET imaging and IPET 3D reconstruction, we reconstruct a total of 14 density maps at a resolution of ∼2 nm from 14 individual double-stranded DNA (dsDNA)-nanogold conjugates. Using these maps as constraints, we derive 14 conformations of dsDNA by projecting a standard flexible dsDNA model onto the observed maps using molecular dynamics (MD) simulations. The variation of the conformations was largely consistent with that from liquid solution, and suggests that the IPET approach provides a most complete experimental determination of flexibility and fluctuation range of these directed nanocrystal assemblies to date. The general features revealed by this experiment can be expected to occur in a broad range of DNA-assembled nanostructures and flexible proteins.

## Results

### Cryo-EM and NS images of DNA-nanogold conjugates

**Figure 1:**
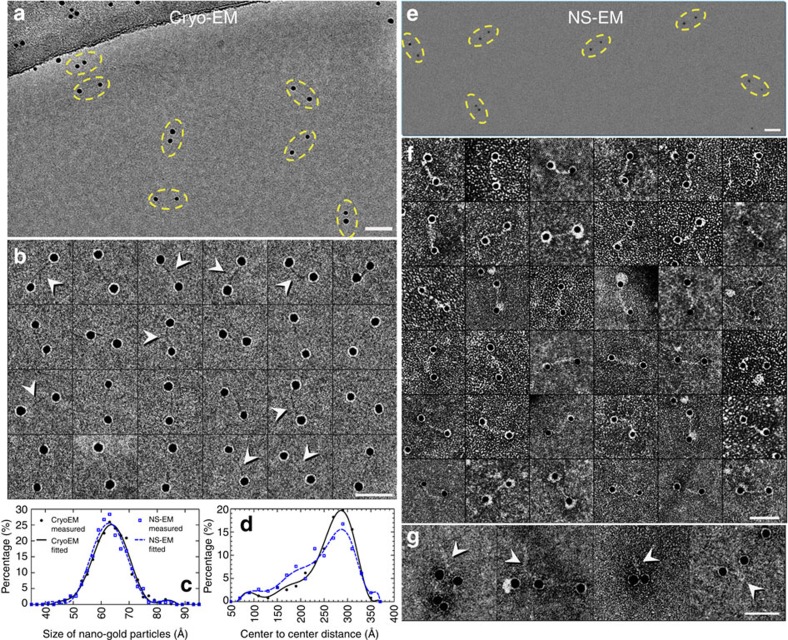
Cryo-EM and OpNS-EM images of dsDNA-nanogold conjugates. (**a**) Cryo-EM images (vitreous buffer, no staining) of 5-nm nanogold particles conjugated to 84-bp dsDNA via a 5′-thiol linker. Pairs of nanogold were marked by yellow dashed ovals. (**b**) 24 representative cryo-EM images of the particles of DNA-nanogold conjugates. The polygonal-shaped areas are the nanogold particles, which were bridged by a fibre-shaped density (high contrast densities were indicated by arrows), ∼20–30 nm in length and ∼2 nm in width. The surfaces of the nanogold particles were coated with a layer of extraneous PEG for surface protection. (**c**) Histogram of the geometric diameters of 1,032 nanogold particles from cryo-EM images and 606 nanogold particles from NS images. (**d**) Histogram of the DNA lengths measured from 516 conjugates from cryo-EM and 303 conjugates from NS. The centre-to-centre length was measured between the centres of each nanogold particle pair. (**e**) NS images and (**f**) 36 representative NS images of the particles. The polygonal-shaped nanogold particles were bridged by a fibre-shaped density, and their surfaces were coated with a layer of extraneous PEG for surface protection. (**g**) A few pairs of nanogold particles were significantly closer in distance to each other, whereas their bridging fabric-like densities were thicker (indicated by arrows), likely due to the supercoiling of the dsDNA. Scale bars=30 nm.

The sample of HPLC-purified 84-bp dsDNA (molecular mass= ∼52 kDa) and two 5-nm nanogold conjugates (Supplementary Fig. 1) were examined by two methods, that is, cryo-EM (native buffer, vitreous ice, no staining) and OpNS^19^,^20^. Cryo-EM, a method often used to prevent artefacts induced by fixatives and stains, can be used to study protein structures under near-native conditions. However, small proteins (<100 kDa) are challenging to be visualized or 3D reconstructed. NS, a historical method, can be used for high-contrast imaging of small proteins through heavy metal salts coating the proteins surfaces^20^,^21^. However, heavy metal reaction can also cause a potential artefact by conventional protocols, including rouleaux formation of lipoproteins^19^,^22^. We used cryo-EM as a control, investigated the parameters related to lipoprotein rouleaux artefact^19^ and reported an OpNS protocol^19^,^22^. The OpNS protocol has been tested by structure-known proteins, including 53-kDa cholesteryl ester transfer protein^23^, GroEL and proteasomes^20^, and the flexible proteins with structure partially known, including immunoglobulin (Ig)-G1 antibody and its peptide conjugates^16^,^17^. The surrounding heavy metal atoms provide more electron scattering and radiation damage resistance than the protein light atoms. In this study, we used cryo-EM and OpNS methods to examine the same sample under -178 °C and room temperature, respectively, (Fig. 1).

Survey cryo-EM and OpNS-EM micrographs (Fig. 1a,e) showed that each pair of nanogold particles was near one another. A statistical analysis of 1,032 nanogold particles from cryo-EM showed that the particles had a diameter of ∼63.5±6.7 Å (mean±s.d.) and a peak population (∼25.1%) diameter of 63.6±1.0 Å (black solid line in Fig. 1c). This measurement is consistent with those from OpNS, that is, 606 nanogold particles from NS micrographs showed a diameter of ∼63.0±6.4 Å (mean±s.d.) and a peak population (∼25.5%) diameter of 62.8±1.0 Å (blue dashed line in Fig. 1c).

To quantitatively identify whether the pairs of nanogold particles are strongly linked together by a statistical method, Pearson's correlation coefficients (PCC)^24^ were used. The PCC, *r*_*x,x*_ (defined as 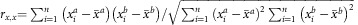 for the *x* axis coordinates of two objects, *a* and *b*) and *r*_*y,y*_ for the *y* axis coordinates were 0.9996 and 0.9976 for cryo-EM, and 0.9984 and 0.9983 for NS-EM results, respectively. The coefficients corresponded well with previous transmission electron microscopy (TEM) observations of the same sample in liquid solution (that is, *r*_*x,x*_=0.934 and *r*_*y,y*_=0.943; ref. 24). The high PCC suggests that the pair of nanogold particles are strongly linked together^24^.

Zoom-in images of 24 representative cryo-EM particle pairs (Fig. 1b) and 36 representative NS particle pairs (Fig. 1f) showed that the polygonal-shaped nanogold particles were bridged by an ∼2-nm-width fibre-shaped density. A statistical analysis of the distances among the 516 pairs of cryo-EM nanogold particles yielded a length of 255.3±48.7 Å (mean±s.d., measured from the centre to centre of the nanogold particles) with a peak population (∼19.7%) distance of 286.4±10 Å (black solid line in Fig. 1d). The distances are similar to that from 303 pairs of NS particles, that is, 245.5±62.6 Å (mean±s.d.) with a peak population (∼15.6%) distance of 287.0±10 Å (blue dashed line in Fig. 1d). Moreover, both lengths were consistent with those measured from liquid solution by small-angle X-ray scattering (SAXS), that is, 28–30 nm (ref. 25) and a standard model of 84-bp dsDNA (∼2 nm wide and ∼30 nm long).

In addition, several pairs of nanogold particles presented abnormally closer to one another in both cryo-EM and NS. The higher-contrast NS images showed that their fibre-shaped bridging densities appeared thicker, but had lengths ranging from ∼20 to 30 nm, thus seeming similar to those of the full-length dsDNA (Fig. 1g). We suspected that these particles may be formed by two conjugates, in which each conjugate lost one nanogold, but met and formed a supercoil via their two dsDNAs. Since the mass is only 52 kDa above that of the regular conjugates, these particles are too small to be identified or isolated by our filtration.

### 3D reconstruction of an individual dsDNA-nanogold conjugate

**Figure 2:**
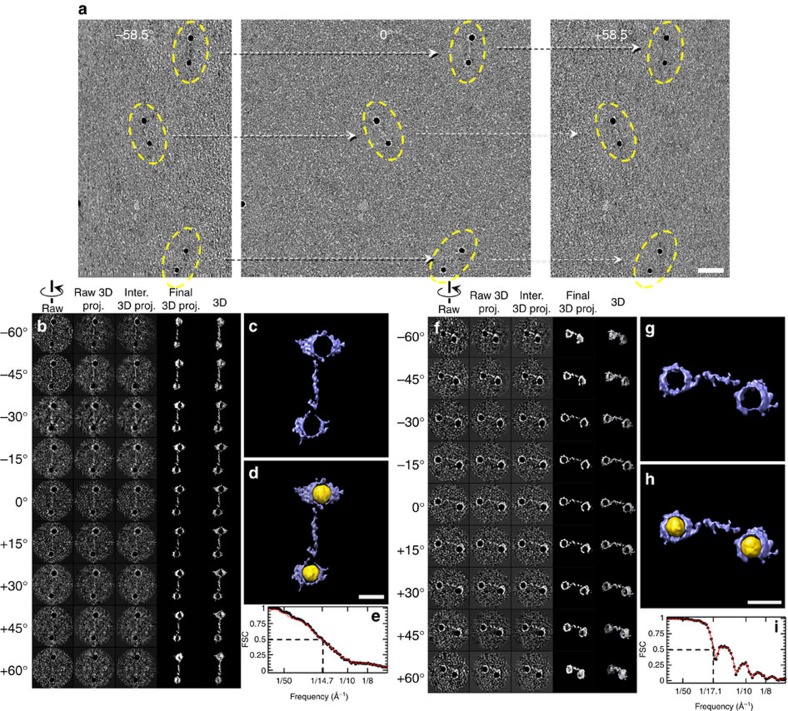
3D reconstruction of two representative DNA-nanogold conjugates by IPET. (**a**) The OpNS samples of DNA-nanogold conjugates were imaged using ET from a series of tilt angles (from −60° to +60° at 1.5° intervals). Three targeted particles (yellow circled) with their orthogonal views are indicated by the linked dashed arrows in the three selected ET tilt micrographs. The relative tilt angles are indicated in each image, and the axis of the tilt is vertical to the images. (**b**) Nine representative tilt images of the first targeted individual particle are displayed in the first column from the left (SNR of DNA portion: ∼0.31). Using IPET, the tilt images (after CTF correction) were gradually aligned to a common centre for 3D reconstruction via an iterative refinement process. The projections of the intermediate and final 3D reconstructions at the corresponding tilt angles are displayed in the next four columns according to their corresponding tilt angles. (**c**) Final IPET 3D density map of the targeted individual particle (SNR of DNA portion: ∼2.44). (**d**) The final 3D density map and its overlaid 3D density maps (final map in blue and its reversed map in gold) indicated the overall conformation of the DNA-nanogold conjugates. (**e**) The FSC analyses under including (black line) and excluding (red line) nanogold portions (two density maps reconstructed from odd and even numbers of tilt images) revealed that the resolutions of the IPET 3D density map were both ∼14.7 Å. (**f**–**i**) The 3D density map of a second individual DNA-nanogold conjugate was reconstructed from the tilt images (SNR of DNA portion: ∼0.56) using IPET. The FSC analysis showed that the 3D reconstruction resolution (SNR of DNA portion: ∼3.26) was ∼17.1 Å. Scale bars=20 nm (**a**) and 10 nm (**d**,**h**).

To obtain a 3D structure of the dsDNA-nanogold conjugates, we used the IPET technique^10^ rather than the conventional single-particle reconstruction method or sub-volume averaging method because these conjugates were not guaranteed to share the same structure (DNA is naturally flexible and dynamic in structure). IPET is used to obtain the *ab initio* 3D structure of an individual particle imaged from a series of tilt angles (Fig. 2a).

Although the DNA portion in the cryo-EM images could be barely visible under a dose of ∼20e^−^ Å^−2^, beyond this dose limitation, the contrasts rapidly disappeared, which prevents us from a full ET data acquisition (∼80 micrographs) and further 3D reconstruction. Thus, we used OpNS-EM for 3D reconstruction.

Although the signal-to-noise ratios (SNRs) of the nanogold portion of ET images (from −60° to +60° at 1.5° increments) were only ∼0.19 to ∼0.41 with an average of ∼0.31, the overall shape of dsDNA was still visible (Fig. 2b and Supplementary Movie 1). After contrast transfer function (CTF) correction, the tilt images were iteratively aligned to a global centre before achieving a final *ab initio* 3D reconstruction (Fig. 2b). During the iterations, SNR of the dsDNA portion gradually increased up to ∼2.44. The final 3D showed an overall handcuff shape (Fig. 2c) at a resolution of ∼14.7 Å, measured based on a Fourier shell correlation (FSC) analysis (details are provided in the Methods section; black line in Fig. 2e). To avoid the potential overestimation of resolution by nanogolds instead of correctly reflecting the resolution of the dsDNA, we masked out the dsDNA portion only to repeat the FSC analyses. The FSC curve was nearly identical to that for nanogolds (red versus black lines in Fig. 2e), suggesting that the resolution is not overestimated by nanogolds.

The nanogold particle surface appeared with a layer of densities, which is possibly thiolated short-chain polyethylene glycol (PEG) molecules used to stabilize the particles against aggregation at high ionic strength (Fig. 2c). Considering that the nanogold particles were in opposite image contrast to the dsDNA, we reversed the image contrast of the final 3D (coloured in gold) and overlaid it with original 3D to display both the dsDNA and nanogold particles in a same 3D map (Fig. 2d). This overlaid map showed the nanogold particles with diameters of ∼73.0 and ∼72.0 Å bridged by a high-density fabric with dimensions of ∼242.0 Å × ∼18.0 Å × ∼18.0 Å (Fig. 2d). The nanogold particle surfaces were surrounded by irregularly shaped densities, the PEG surface protection layer.

**Figure 3:**
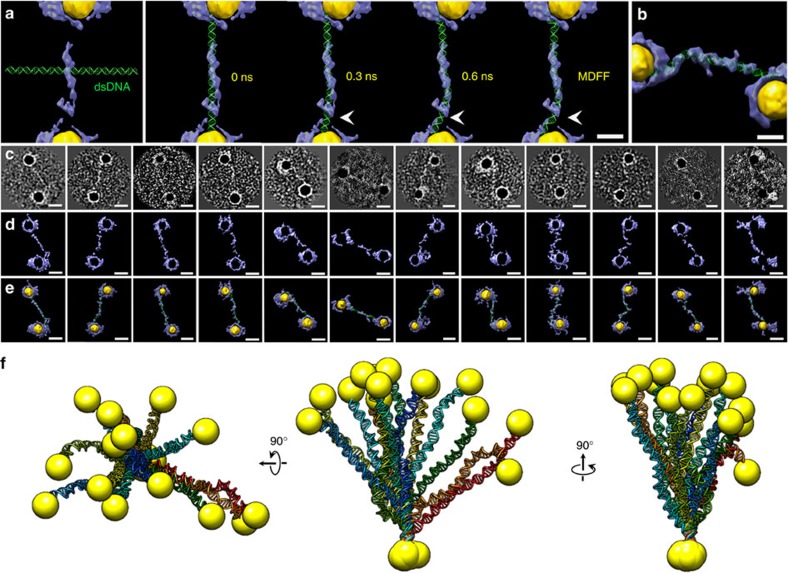
3D conformations of 14 dsDNA structures. Conformations obtained by flexibly fitting the dsDNA model onto the EM density maps using targeted MD simulations. (**a**) The final density map provided a constraint for the TMD simulations to achieve a new DNA conformation. Four snapshot images during the TMD simulation illustrate the process of flexibly docking the DNA model into the IPET density map to achieve a new conformation of DNA (arrow indicated the great conformational changing portion of DNA). During this process, the DNA conformation was allowed to change its structure while maintaining its chemical geometry and bonds with local energy minimization. (**b**) The final conformation of the second dsDNA structure was obtained from the second density map by following the same processes. (**c**–**e**) Gallery of 12 additional conformations from the 3D density maps of an additional 12 DNA-nanogold conjugates reconstructed using IPET. (**c**) Selected projections of the 3D density map of each individual DNA-nanogold conjugate. (**d**) Final 3D density maps of the individual DNA-nanogold conjugates. (**e**) The overlaid density maps of the final 3D reconstruction (grey) and its reversed contrast map (gold) revealed the overall conformation of the DNA-nanogold conjugates. A standard 84-bp dsDNA structure was flexibly docked into each density map to achieve the new conformations via TMD simulations. (**f**) Conformational flexibility and dynamics of the DNA-nanogold conjugate. Fourteen conformations of the DNA-nanogold conjugates were aligned together based on their first 14 bp. The distribution of dsDNA is shown from three orthogonal views. Scale bars=5 nm (**a**,**b**) and 10 nm (**c**–**e**).

Although the 3D resolution was insufficient to determine the structure of the dsDNA, the overall shape could be used as a constraint to flexibly dock a standard structure of an 84-bp dsDNA into it to achieve a dsDNA conformation. By satisfying both the best fit to the density map and the chemical minimal energy requirements, we obtained a dsDNA conformation via gently bending the straight dsDNA structure into the density map using the CHARMM force field for all MD simulations^26^,^27^ (Fig. 3a and Supplementary Movie 1).

By repeating the above process, we reconstructed 3D from second individual dsDNA-nanogold conjugate (Fig. 2f–i). The representative tilt images showed that the dsDNA was still visible (Fig. 2f), and the SNR of dsDNA portions ranged from ∼0.41 to ∼0.62, with an average of 0.56. Through IPET reconstruction, the tilt images were aligned to a global centre (Fig. 2f), and the SNR was increased up to ∼3.26 (Fig. 2g). The final 3D reconstruction at ∼17.1-Å resolution (based on the FSC analyses with and without the nanogold portion conditions; Fig. 2i) showed a handcuff shape. The overlaid density map (the final 3D map with its reversed map, coloured in gold) showed that the nanogold particles had diameters of ∼71.0 and ∼65.0 Å bridged by a fabric-like density with dimensions of ∼191.0 Å × ∼20.0 Å × ∼20.0 Å (Fig. 2h). Again, the nanogold particles were surrounded by irregularly shaped densities of a PEG surface protection layer. By flexibly docking the standard dsDNA structure into the bridging portion and following MD simulation for energy minimization, a second conformation of dsDNA was obtained (Fig. 3b).

### 3D reconstructions of another 12 DNA-nanogold conjugates

Through particle-by-particle 3D reconstructions, additional 12 conjugates were reconstructed using IPET (Fig. 3, Supplementary Figs 2–13 and Supplementary Table 1). The selected projections of the final 3D reconstructions showed a fabric DNA density between two nanogold particles (Fig. 3c). The overlaid density maps (reversed maps are coloured in gold; Fig. 3d,e) also confirmed that the nanogold particles were connected by densities attributable to dsDNA. Although up to ∼30% of the fabric-like densities in those maps (Fig. 3e) could not be fully observed under the selected contour levels owing to various factors, such as uneven staining, image noise and reconstruction errors, these defects have a limited effect on the spacing distribution and the overall shape determination of the dsDNA due to its connectivity. The resolutions of ∼20 Å still allowed us to flexibly dock the dsDNA model to obtain 12 additional DNA conformations via MD simulations (Fig. 3e and Supplementary Figs 2c–13c).

### Statistical analyses of DNA-nanogold conjugates structures

Aligning the 14 conformations of dsDNA along their first 14 bp yielded a distribution in a shape of a bundle of flowers (Fig. 3f). Considering that the 14 conformations is insufficient to reveal the full 3D distribution of DNA conformations, only 1D distribution analysis was conducted, that is, the nanoparticle size and DNA length. The histogram of the nanogold particle sizes measured from the 3D reconstructions (the measurement method is described in Methods section) showed that the geometric mean of the nanoparticle diameters was 65.7±5.0 Å, which is similar to the diameter measured from the 2D images of 606 nanogold particles (62.8±1.0 Å at peak population, ∼25.5%; Fig. 1d and Supplementary Table 1). The average distance measured from the 3D reconstructions (the measurement method is described in Methods section) was 291.1±31.9 Å (mean±s.d.), which was longer than the mean distance measured from the 2D images (245.5±62.6 Å), but was similar to the distance of 287.0±10.0 Å at the peak population (∼15.6%; Fig. 1d). Approximately 69% of the distances was shorter than the peak distance, whereas ∼31% were longer (Supplementary Table 1). This uneven distribution of the distance around the distance of the peak population is likely owing to the portion of dsDNA that formed a supercoiled structure but were not 3D reconstructed or measured. These variations reflected the conformational flexibility and dynamics of the dsDNA between the two conjugated nanogold particles.

Although the particles were flash-fixed by stain heavy metals and attached to a substrate (carbon film, which may cause certain artefacts, such as a preferred orientation, flatness and an uneven staining distribution), the statistical analyses showed that the measured lengths from 303 dimers were highly similar to the same sample measured in solution (Supplementary Table 2; refs 24, 25). In detail, the distances measured using SAXS from the same sample in solution were ∼280 Å on average and ∼320 Å at the peak population^25^, which were ∼10–15% longer than those measured from our 2D images (Fig. 1d). Considering that the length measured from 2D images corresponded to the projection distance of the 3D length in solution, the 2D projection is naturally shorter than the length in 3D by a factor of *π*/4 under an isotropic distribution assumption condition. Based on the solution measured distances, their corresponding 2D projection distances should be ∼220 Å (*π*/4 × 280 Å) on average and ∼250 Å (*π*/4 × 320 Å) at the peak population, which were ∼10–12% shorter than our measurement from 2D images. Our measured lengths (∼246 and ∼287 Å) were between the 3D lengths (∼280 and ∼320 Å) and the 2D projection lengths (∼220 and ∼250 Å), suggesting that our particles have a certain preferred orientation to the carbon film. The mean distance measured in solution via *in situ* TEM was ∼180 Å (ref. 24), which was ∼26% shorter than the mean measured from our 2D images. Although the short-distance views of the targeted conjugate was specifically chosen for easy tracking and imaging in the *in situ* experiment, the mean value was still close to the error bar range in our measurement.

### Analyses of bending energy of DNA-nanogold conjugates

The conformations of dsDNA provide an opportunity to study the DNA bending energy. The bending energy can be calculated based on a simple worm-like chain (WLC) model^25^,^28^,^29^, in which the energy of dsDNA is simplified to the bending potential while ignoring the distortion potential at the single-base pair level. The calculation of the bending energy depends on the local bending angles of each base pair, which is governed by a single parameter for the mechanical response, that is, the persistence length. Although the persistence length could be extracted by measuring the tangent correlation function for a WLC, the length of our 84-bp DNA here is too short to derive a persistence length. Thus, the widely used persistence length of 50 nm was used to compute the bending energy as this length is a favourable parameter to describe the dsDNA conformational statistics for constructs composed of tens of base pairs^25^.

**Figure 4:**
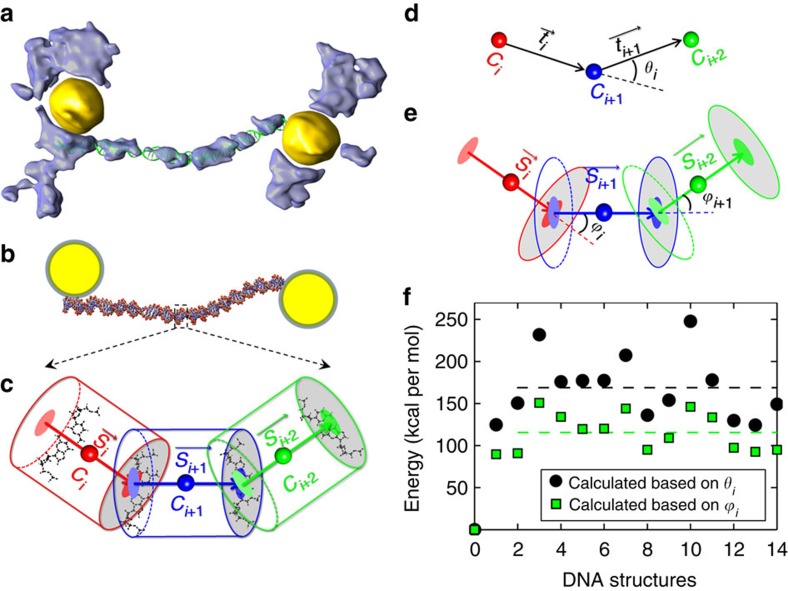
Bending energy distribution of dsDNA. (**a**) dsDNA conformation was obtained by fitting the standard dsDNA model into the IPET 3D density map. (**b**) Schematic model illustrating that the nanogold interacts with the dsDNA and that the dsDNA contains kink regions that carry bending elasticity. (**c**) Cylinder model illustrating the bending angles between two connected base pairs. The cylinder is defined by the two consecutive dsDNA base pairs. (**d**) The bending angle can be presented by the angle *θ*_*i*_, formed by the centres of three consecutive cylinders, or (**e**) by the angle *ϕ*_*i*_, formed by the centre axes of two consecutive cylinders. (**f**) Based on the two types of measured angles, *θ*_*i*_ and *ϕ*_*i*_, the bending energies for each DNA conformation were calculated and plotted based on a simple WLC model. The averaged bending energies from the two types of angles are indicated by the dashed lines. The bending energy of the standard DNA model was also calculated and indicated as structure no. 0 as a control.

To measure the local bending angle (Fig. 4a), each of two nearby base pairs was first fitted with a small standard cylinder with a fixed length and width (Fig. 4b,c). Two types of angles can be defined to represent the bending angle of the base pair, that is, the angle *θ*_*i*_, formed between centre-to-centre directions of three nearby cylinders (Fig. 4d), and the angle *ϕ*_*i*_, formed between the two axes of nearby cylinders (Fig. 4e). The energies of each base pair were calculated and summed to represent the total energy of this dsDNA conformation. The energy distribution of the 14 conformations showed that the averaged bending energies were ∼116 and ∼169 kcal mol^−1^ based on two types of angle definition (Fig. 4f and Supplementary Table 2). The average bending energies were ∼2–3 times higher than the bending energy calculated based on a theoretical WLC model prediction on 84-bp DNA (∼50 kcal mol^−1^ at room temperature)^25^, suggesting that the 14 DNA conformations were more flexible than the prediction.

### Parameters influence the length and bending energy of DNA

Above analyses showed two definition types of bending angles, that is, *θ*_*i*_ and *ϕ*_*i*_, which could cause a ∼50% difference on the computed bending energy from the experimental DNA conformations. Considering that the EM observed DNA is more flexible than predicted, it is worthy to evaluate whether other factors may also influence the measurement of the DNA length and bending energy, such as the fluctuation of distal ends, DNA modelling methods, noise bias in the EM density map, initial model bias and temperature. Additional analyses were performed as the following: (i) the bending energy based on the central 42 bp was recalculated. This was because 84-bp DNA is relatively short and the ends of the chain exhibit greater fluctuations than the middle portion. The results obtained from the calculation showed that the bending energy is ∼15% less than when using all 84 bp (Supplementary Table 2), suggesting that DNA is still more flexible than the WLC prediction. (ii) The initial model of DNA was obtained by manually fitting a standard DNA model to each EM configuration. Considering that the manual operation may lead to kinks, which are difficult to be repaired by MD simulation, we used another method to generate a smooth curved model of DNA whose structure was as close as possible to the canonical B-form DNA double helix (details are in Methods section). Before conducting any simulation, we calculated its bending energy, which is only ∼10% of the above bending energy, and only ∼20% of the WLC prediction, suggesting that the smooth DNA is more stiff than the WLC prediction, and EM configurations. (iii) After submitting this smooth model for energy minimization using the nanoscale molecular dynamics version 2 (NAMD2) software package^30^, we found that the bending energy was increased ∼4–5 times, thus nearly approaching the WLC prediction. (iv) However, after submitting the model to further MD simulations for only 0.1 ns, the calculated energy jumped up to ∼80% of the energy from the EM configuration, thereby confirming that the DNA is more flexible than the WLC prediction. This result suggests that the MD simulations may be the key role to increase the bending energy and result in a more flexible DNA model. (v) To evaluate how the noise in the density map may influence the bending energy, we conducted MD flexible fitting (MDFF) based on the smooth model to constrain its structure with the EM density map. The calculated bending energy immediately jumped up to an even higher level, that is, ∼10% more than that from the EM configurations. This test suggests that the MDFF and noise in the EM density could be critical in causing the DNA model to be more flexible than it should be. (vi) To avoid a potential influence to the energy from the given initial models, a standard and straight model of DNA was used for MD simulations under the same condition, that is, under 0.15 M physiological salt solution, temperature of 298 K and a pressure of 1 atm. Using NAMD2 for 20 ns of simulation without any constraint for DNA conformational changes, the equilibration of DNA in a box of ∼347.3 Å × ∼93.4 Å × ∼50.8 Å was monitored using the root mean square deviation by visual MD (VMD)^31^ (Supplementary Fig. 14a). The bending energies (Supplementary Fig. 14b) and the distances between the two distal ends (Supplementary Fig. 14c) revealed that the system became nearly balanced after 8 ns. The statistical analyses of the bending energies of the DNA in its last 10-ns simulations showed that the average energy was 99.1±10.9 kcal mol^−1^ with a peak population (∼7.1%) energy of 97.4±1.0 kcal mol^−1^ based on the bending angles of the *ϕ*_*i*_ calculation, whereas the average energy was 152.1±16.1 kcal mol^−1^ with a peak population (∼4.6%) energy of 151.3±1.0 kcal mol^−1^ based on the bending angles of the *θ*_*i*_ calculation (Supplementary Fig. 14b,d). This energy is surprisingly similar to that of the EM configuration (∼10–15% lower) (Supplementary Table 2). The statistical analyses of the length between the two distal ends of equilibrated DNA in the last 10 ns of the simulations showed that the average length was 268.5±2.1 Å with a peak population (∼18.5%) length of 267.9±0.5 Å (Supplementary Fig. 14c,e). The distance is similar to the length at the peak population measured from TEM images. (vii) To evaluate how temperature influences the bending energy measurement using MD simulations, the above processes were repeated under a higher temperature, that is, 310 K instead of 298 K (Supplementary Fig. 15). The bending energies calculated under the higher temperature in the last 10 ns showed that the average energy was 120.8±14.7 kcal mol^−1^ with a peak population (∼6.1%) energy of 115.1±1.0 kcal mol^−1^ based on the bending angles of the *ϕ*_*i*_ calculation, whereas the average energy was 177.9±15.6 kcal mol^−1^ with a peak population (∼5.1%) energy of 177.5±1.0 kcal mol^−1^ based on the bending angles of the *θ*_*i*_ calculation (Supplementary Fig. 15b,d). The energy is increased by ∼20% from those under 298 K and becomes more similar to that of the EM configuration, suggesting that the temperature is related, but not critical. The statistical analyses on the length showed that the averaged length was 269.5±2.9 Å with a peak population (∼13.3%) length of 271.1±0.5 Å (Supplementary Fig. 15c,e), which is similar to those measured from the EM configurations and 298 K MD simulation, suggesting that the length is insensitive to the temperature. (viii) To further confirm that the length is insensitive to bending energy, one EM configuration with the DNA length of ∼241.0 Å was performed by MD simulations under a length constrain (Supplementary Fig. 16). This length is close to the mean length of the DNA portion estimated from solution using SAXS^25^. The bending energy in the last 10 ns was 105.8±10.7 kcal mol^−1^ with a peak population (∼7.3%) energy of 102.7±1.0 kcal mol^−1^ based on the bending angles of the *ϕ*_*i*_ calculation, whereas the average energy was 163.6±17.4 kcal mol^−1^ with a peak population (∼5.0%) energy of 158.4±1.0 kcal mol^−1^ based on the bending angles of the *θ*_*i*_ calculation (Supplementary Fig. 16b,c). This energy is similar to that of the other EM configurations and simulations, suggesting that the length cannot reflect the bending energy and flexibility of DNA.

Above tests showed, although EM density maps contain noise, that the bending energy can be influenced limitedly by the initial models and the EM map configuration. The similar bending energies calculated from different MD simulations and initial models suggested that the DNA is more flexible than WLC prediction. However, we cannot exclude those MD simulations that may result in DNA, which presents more flexibility than WLC prediction.

## Discussion

Although the direct imaging of dsDNA has been previously reported using heavy metal shadowing^32^,^33^ and NS methods^34^,^35^,^36^, to the best of our knowledge, the 3D structure of an individual dsDNA strand has not previously been achieved. It has been thought that individual dsDNA would be destroyed under the high energy of the electron beam before a 3D reconstruction, or even a 2D image, is able to be achieved. Our NS tilt images showing fibre-shaped dsDNA bridging two conjugated nanogold particles demonstrated that the dsDNA can in fact be directly visualized using EM, which is consistent with the recently reported single-molecule DNA sequencing technique via TEM^36^. The resolutions of our density maps ranged from ∼14 to ∼23 Å, demonstrating that an intermediate-resolution 3D structure can be obtained for each individual macromolecule. This capability is consistent with our earlier report of a ∼20-Å resolution 3D reconstruction of an individual IgG1 antibody using the same approach^16^,^17^.

Notably, a total dose of ∼2,000e^−^ Å^−2^ used in our ET data acquisition is significantly above the limitation conventionally used in cryo-EM (∼80–100e^−^ Å^−2^), which can be suspected to have certain artefact from radiation damage. In cryo-EM, the radiation damage could cause sample bubbling, deformation and knockout effects; in NS, only the knockout phenomena is often observed, in which the protein is surrounded by heavy atoms that were kicked out by electron beam. Since the sample was coated with heavy metal atoms and were dried in air, the bubbling and deformation phenomena were not usually observed. The heavy metal atoms that coat the surface of the biomolecule can provide a much higher electron scattering than from a biomolecule only inside lighter atoms. The scattering is sufficiently high to provide enough image contrast at our 120-kV high tension; thus, a further increase to the scattering ability by reducing the high tension to 80 kV may not be necessary for this NS sample. In addition, the heavy atoms can provide more radiation resistance and allow the sample to be imaged under a higher dose condition. The exact dose limitation for NS is still unknown. The radiation damage related artefact in NS samples is knockout, which could reduce the image contrast and lower the tilt image alignment accuracy and 3D reconstruction resolution. In our study, a total dose of 2,000e^−^ Å^−2^ did not cause any obvious knockout phenomena, but provides a sufficiently high contrast for the otherwise barely visible DNA conformations in each tilt series. The direct confirmation of visible DNA in each tilt image is essentially important to us to validate each 3D reconstruction, especially considering this relatively new approach.

Our 3D reconstruction algorithm used an *ab initio* real-space reference-projection match iterative algorithm to correct the centres of each tilt images, in which the equal tilt angle step for 3D reconstruction of a low contrast and asymmetric macromolecule was used. This method is different from recently reported Fourier-based iterative algorithm, termed equally sloped tomography, in which the pseudo-polar fast Fourier transform, the oversampling method and internal lattice of a targeted nanoparticle are used to achieve 3D reconstruction at atomic resolution^37^.

It is generally challenging to achieve visualization and 3D reconstruction on an individual, small and asymmetric macromolecule by other conventional methods; our method demonstrated its capability for 3D reconstruction of 52 kDa 84-bp dsDNA through these studies: IgG1 antibody 3D structural fluctuation^17^, peptide-induced conformational changes on flexible IgG1 antibody^5^, floppy liposome surface binding with 53-kDa proteins^38^, all of which suggest that this method could be used to serve the community as a novel tool for studying flexible macromolecular structures, dynamics and fluctuations of proteins, and for catching the intermediate 3D structure of protein assembling.

DNA-based self-assembling materials have been developed for use in materials science and biomedical research, such as DNA origami designed for targeted drug delivery. The structure, design and control require feedback from the 3D structure, which could validate the design hypothesis, optimize the synthesis protocol and improve the reproducible capability, while even providing insight into the mechanism of DNA-mediated assembly.

## Methods

### Synthesis of DNA-nanogold conjugates

Conjugation of DNA-nanogold was synthesized according to a previously published procedure^1^. In brief, ∼5.5-nm nanogold particles were stabilized via exchanging with bis-(*p*-sulfonatophenyl) phenylphosphine. DNA sequences modified with a 5′-thiol moiety were purified via polyacrylamide gel electrophoresis. DNA thiolated at the 5′ end was re-suspended in buffer (10 mM Tris pH 8 and 0.5 mM EDTA). Nanogold particles and DNA were combined at a stoichiometric ratio of 1:2 in the presence of a reducing agent. The formed monoconjugates were separated using anion-exchange HPLC, and the fractions were concentrated using an Amicon Ultra spin filter, MW=100,000 (EMD Millipore Corp., Billerica, MA, USA). Twenty microlitres of nanogold monoconjugates, each containing complementary strands of DNA, were combined stoichiometrically, as determined by absorption at 520 nm, and were allowed to react overnight at room temperature^7^. The final conjugates were purified from unreacted monoconjugates via agarose gel electrophoresis.

### Sequences

84-Base DNA sequence. 5′-thiol-CCGGCGGCCCAGGTGTATCAGTGTTCGTTGCAAGCTCCAACATCTGAGTACCACGCATACTATACTTGAAATATCCGCGCCCGG-3′.

84-Base DNA complement. 5′-thiol-CCGGGCGCGGATATTTCAAGTATAGTATGCGTGGTACTCAGATGTTGGAGCTTGCAACGAACACTGATACACCTGGGCCGCCGG-3′.

### Preparation of cryo-EM and OpNS-EM specimens

The cryo-EM specimens were prepared as described previously^39^. In brief, an aliquot (∼4 μl) of DNA-nanogold sample at a concentration of ∼20 μg ml^−1^ was placed on a glow-discharged lacey carbon film-coated copper grid (Cu-200LC, Pacific Grid-Tech, San Francisco, CA, USA). The samples were blotted with filter paper from both sides at ∼90% humidity and 4 °C with a Leica EM GP rapid-plunging device (Leica, Buffalo Grove, IL, USA) and then flash-frozen in liquid ethane. The flash-frozen grids were transferred into liquid nitrogen for storage. The NS specimens were prepared by OpNS protocol as described previously^19^,^22^. In brief, an aliquot (∼4 μl) of DNA-nanogold sample at a concentration of ∼20 μg ml^−1^ was placed on a thin carbon-coated 200-mesh copper grid (Cu-200CN, Pacific Grid-Tech; CF200-Cu, Electron Microscopy Sciences, Hatfield, PA, USA) that had been glow-discharged. After ∼1 min of incubation, the excess solution was blotted with filter paper. The grid was then washed with water and stained with 1% (w/v) uranyl formate on Parafilm before air-drying with nitrogen^19^,^22^.

### TEM un-tilted data acquisition and imaging process

Both cryo-EM and OpNS samples were examined using a Zeiss Libra 120 Plus TEM (Carl Zeiss NTS) operating at 120 kV high tension with 20 eV in-column energy filtering at −178 °C (for cryo-EM) and room temperature (for NS). A Gatan 915 cryo-holder was used. The micrographs were acquired using a Gatan UltraScan 4K × 4K CCD under a magnification of 20–125 kx (each pixel of the micrographs corresponded to ∼0.59 to ∼0.094 nm in specimens) under a dose of ∼5–15e^−^ Å^−2^ (for cryo-EM) and ∼40–90e^−^ Å^−2^ (for NS-EM), and defocus of 0.2–3 μm (for cryo-EM) and <1.0 μm (for NS-EM). The defocus and astigmatism of each micrograph were examined using EMAN *ctfit* software^40^ after the X-ray speckles were removed. The micrographs with distinguishable drift effects were excluded. The remaining micrographs were filtered using a Gaussian boundary high-pass filter within a resolution range of ∼200–500 nm.

The statistical analysis of the diameter of the nanogold particles was based on the geometric mean^22^ of 1,032 cryo-EM nanogold particles and 606 NS nanogold particles. Each particle was measured based on two perpendicular directions in which one of the diameters was measured along the longest direction of the particle^22^. The DNA lengths were measured by calculating the distance between the centres of two nanogolds (centre-to-centre distance). The lengths of 516 pairs from cryo-EM images and 303 pairs from NS images were measured and then submitted for histogram analyses by using Matlab software.

### ET data acquisition and image pre-process

The TEM holder was tilted at angles ranging from −60° to +60° at 1.5° increments and controlled using Gatan tomography software that was pre-installed in the microscopes (Zeiss Libra 120 TEM). The TEM was operated under 120 kV with a 20 eV energy filter. The tilt series were acquired using a Gatan Ultrascan 4K × 4K CCD camera under low-defocus conditions (<1.0 μm) and a magnification of 125 kx (0.094 nm per pixel). The total electron doses were ∼1,500–2,500e^−^ Å^−2^. The micrographs were initially aligned together using the IMOD software package^41^. The CTF was then corrected using TOMOCTF^42^. The tilt series of the particles in square windows of 512 × 512 pixels (∼48 nm) were semi-automatically tracked and windowed using IPET software^10^.

### IPET 3D reconstruction

In the IPET reconstruction process^10^, a tilt series of CTF-corrected images containing a single DNA-nanogold particle (in size of ∼48 nm) was directly back-projected into an *ab initio* 3D density map as an initial model based on their corresponding goniometer tilt angles. The refinement was started using this initial model to align each tilt image via translational alignment to the projections of the initial model. During the refinement, automatically generated filters and a circular-shaped mask with a Gaussian boundary were sequentially applied to the tilt images and references to increase the alignment accuracy^10^. The resolution was defined based on FSC in which the aligned images were split into two halves based on an odd- or even-numbered index to generate two 3D reconstructions for computing their FSC curve against the spatial frequency shells in Fourier space. The frequency at which the FSC curve first falls to a value of 0.5 was used to represent the resolution of the IPET 3D density map. All of the IPET density maps presented in the figures were low-pass filtered to 16–20 Å. The DNA portion SNR in each 3D map was calculated using the equation SNR_*y*_=(*I*_s_*−I*_b_*)/N*_b_, where *I*_*s*_ is the average power inside the particle, *I*_*b*_ is the average power outside the particle (nanogold area was excluded), and *N*_b_ is the s.d. of the noise calculated from the background s.d. outside the particle (nanogold area was excluded). The particle area was defined using a particle-shaped mask generated from the IPET final 3D with low-pass filtering to ∼25–30 Å and set as three times its molecular weight. This same method was used to calculate the 2D SNR. The 2D mask was generated based on the 3D projection at each tilt angle.

### Conformation of DNA

A standard model of the 84-bp double-helix B-form DNA (dsDNA) was generated using the online DNA sequence to structure tool^43^. By comparing the standard model to the density map, we aligned the best-fit positions onto the EM density map envelope using Chimera^44^. Using the best-fit positions as the target markers, we drove the DNA conformational change of the model to agree with the DNA portion in the 3D density map with a pre-calculated force via a targeted MD (TMD) simulation^45^. After 0.6 ns of TMD simulation, the DNA was moved onto the density map, and we further equilibrated the DNA conformation in a water solution at room temperature via a MDFF simulation under the constraint of the original density map while protecting the secondary structure. Both TMD and MDFF simulations were performed using NAMD2 software package from the University of Illinois at Urbana-Champaign^30^.

A method to generate a smooth DNA model to match the EM configuration was conducted as follows: the DNA portion between two nanogolds was first marked out by a series of consecutive 3D points. Labelled points were automatically fitted with a smooth quadratic or cubic Bézier curve by GraphiteLifeExplorer software^46^. A DNA model was generated to be as close as possible to the curve with a canonical DNA helix. For comparison, the model was also submitted for refinement by an energy minimization with NAMD2 at 0.15-M salt concentrations. The energy minimization was last for 30,000 steps and followed by 0.1-ns all-atom simulation. For a further comparison, the model was also constrained by the electron density map using MDFF. All the MD simulations were performed using CHARMM force field^26^,^27^.

### Calculation of dsDNA bending energy

To calculate the DNA bending energy, we measured the local bending angles, as illustrated in Fig. 4c–e. In detail, the two nearby bases of the standard DNA model were rigidly aligned to the derived dsDNA conformations using VMD^31^. Based on the centres (*C*_*i*_) and centre directions (indicated as a vector 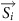) of the aligned standard DNA bases, whose orientations are represented by a small cylinder, the two types of DNA bending angles were measured, that is, the angle *θ*_*i*_ formed by the centres of three consecutive cylinders and the angles *ϕ*_*i*_ between the central axes of two consecutive cylinders. Therefore, the DNA bend energy *E*_bend_ was calculated according to 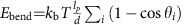
or 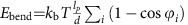, where *l*_p_ is the dsDNA persistence length (50 nm) and *d* is the distance between the base pairs (3.4 Å; ref. 25).

### MD simulation of dsDNA bending energy and length in solution

The MD simulation analyses were performed according to the following three steps: (i) a standard model of 84-bp dsDNA was embedded into a cubic box that extended at least 15 Å away from the DNA surface. The box contained a total of 49,679 TIP3P water molecules, 307 Na^*+*^ atoms and 141 Cl^−^ atoms to simulate the 0.15-M physiological salt concentration and was constructed using VMD^31^. The system, which contained a total of 154,813 atoms, was subjected to energy minimization via 20,000 steps to remove the atomic clashes using the NAMD2. The DNA backbone atoms were fixed in the first 10,000 steps. In the second 10,000 steps, the DNA backbone atoms were constrained under a force constant of 5 kcal mol^−1^ Å^−2^. The energy-minimized system was subsequently heated from 0 to 298 K over 120 ps to initiate the 20-ns all-atom MD simulations. The systems under 1 atm and 298 K reached equilibration after 10 ns; the DNA backbone constraints were removed after the first 0.2 ns of the simulations. During the simulations, the temperature was maintained via Langevin dynamics^47^ with a damping coefficient of 5 ps, and the 1 atm pressure was maintained using the Langevin piston Nose–Hoover method^48^ with a piston period of 100 fs and a decay time of 50 fs. Periodic boundary conditions and a cutoff distance of 12 Å for van der Waals' interactions were applied, and the Particle–Mesh Ewald method^49^ with a grid spacing of <1 Å was used to compute the long-range electrostatic interactions. (ii) The above system was submitted again under a higher balanced temperature of 310 K instead of 298 K. (iii) The 11th dsDNA conformation derived from the previous flexible docking was embedded into a cubic box containing 52,093 TIP3P water molecules, 313 Na^+^ atoms and 147 Cl^−^ atoms. The criterion to select this conformation as a representative conformation of the dsDNA model was a length of ∼241.0 Å, which was close to the mean length of the DNA estimated from solution by SAXS^25^. The system, which contained 162,067 atoms, was subjected to energy minimization via 10,000 steps to remove the atomic clashes using NAMD2. The energy-minimized system was subsequently heated from 0 to 293 K over 60 ps to initiate the 20-ns all-atom MD simulation, in which the end-to-end distance of this DNA was constrained at 241.0 Å under a force constant of 50 kcal mol^−1^ Å^−2^. This system under 1 atm and 293 K reached equilibration after 10 ns of simulation. In the above processes, the bending energies and length of the dsDNA conformations in the last 10 ns were submitted to the statistical analyses.

## Additional information

**Accession codes:** TEM 3D density maps of 14 DNA-nanogold conjugates are available from the EM data bank as EMDB IDs 2948–2961.

**How to cite this article:** Zhang, L. *et al.* Three-dimensional structural dynamics and fluctuations of DNA-nanogold conjugates by individual-particle electron tomography. *Nat. Commun.* 7:11083 doi: 10.1038/ncomms11083 (2016).

## Supplementary Material

Supplementary Information Supplementary Figures 1-16 and Supplementary Tables 1-2

Supplementary Movie 1 Individual particle electron tomography (IPET) 3D reconstruction of an individual DNA-nanogold conjugate. A tilt series of the electron microscopic survey views of an individual molecule containing two 5.5-nm nanogold particles conjugated with 84-base-pair dsDNA, was prepared via optimized negative staining (OpNS). The higher-magnification views of an individual DNA-nanogold conjugate particle show its detailed structure. Via the IPET 3D reconstruction, the tilt images of the targeted conjugates were aligned to a global center and reconstructed onto a 3D density map. By overlaying the 3D density map (in gray) with its inverted map (in gold), the resulting map showed the structure of the DNA nanogold conjugate. By flexibly docking a standard dsDNA model into the fabric density portion via targeted molecular dynamics (TMD) simulation, a new conformation of dsDNA and nanogold was achieved. We revealed the flexibility of the DNA-nanogold conjugates by aligning a total of 14 structures of the DNA-nanogold conjugates.
