# Supplementary material for: Three-dimensional structural dynamics and fluctuations of DNA-nanogold conjugates by individual-particle electron tomography
Source: Nat Commun. 2016 Mar 30;7:11083. doi: 10.1038/ncomms11083 (PMC4820932; doi:10.1038/ncomms11083)
Supplement: Supplementary Information — Supplementary Figures 1-16 and Supplementary Tables 1-2 [file ncomms11083-s1.pdf]

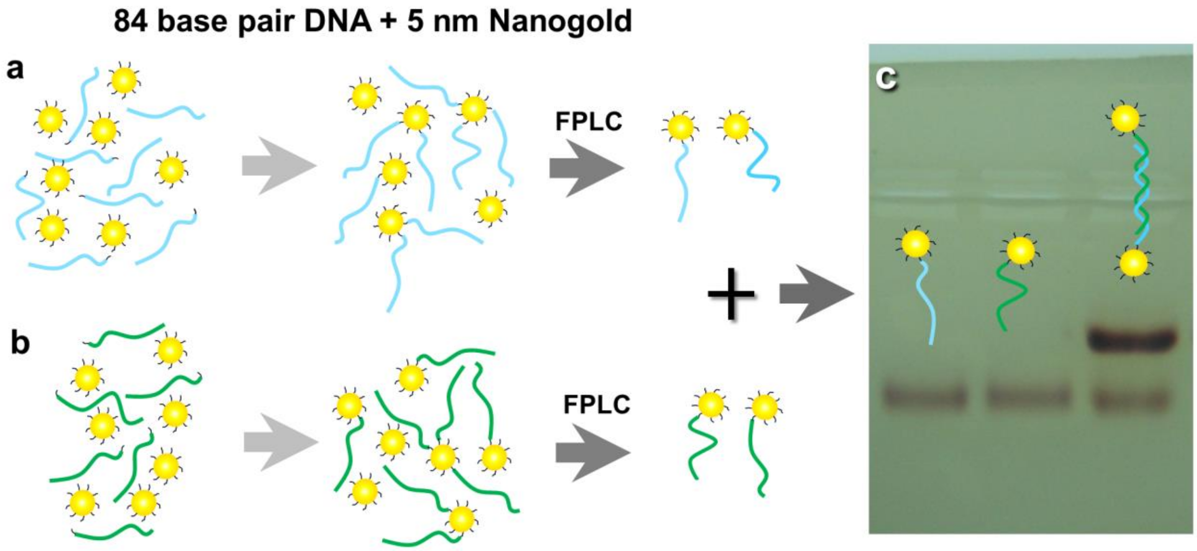

Supplementary Figure 1 | Schematic diagram of the synthesis of DNA-nanogold conjugates. (a and **b)** The 5.5-nm nanogold particles, stabilized via exchange with bis-(*p*-sulfonatophenyl) phenylphosphine (BSPP), were incubated with 5' thiol-modified cDNA sequences at a stoichiometric ratio of 1:2. The monoconjugates were separated via anion-exchange high-performance liquid chromatography (HPLC). (**c**) The conjugates containing complementary strands of DNA were combined stoichiometrically to react overnight at room temperature, as determined by absorption at 520 nm. The dimers were purified from unreacted monoconjugates using HPLC and agarose gel electrophoresis.

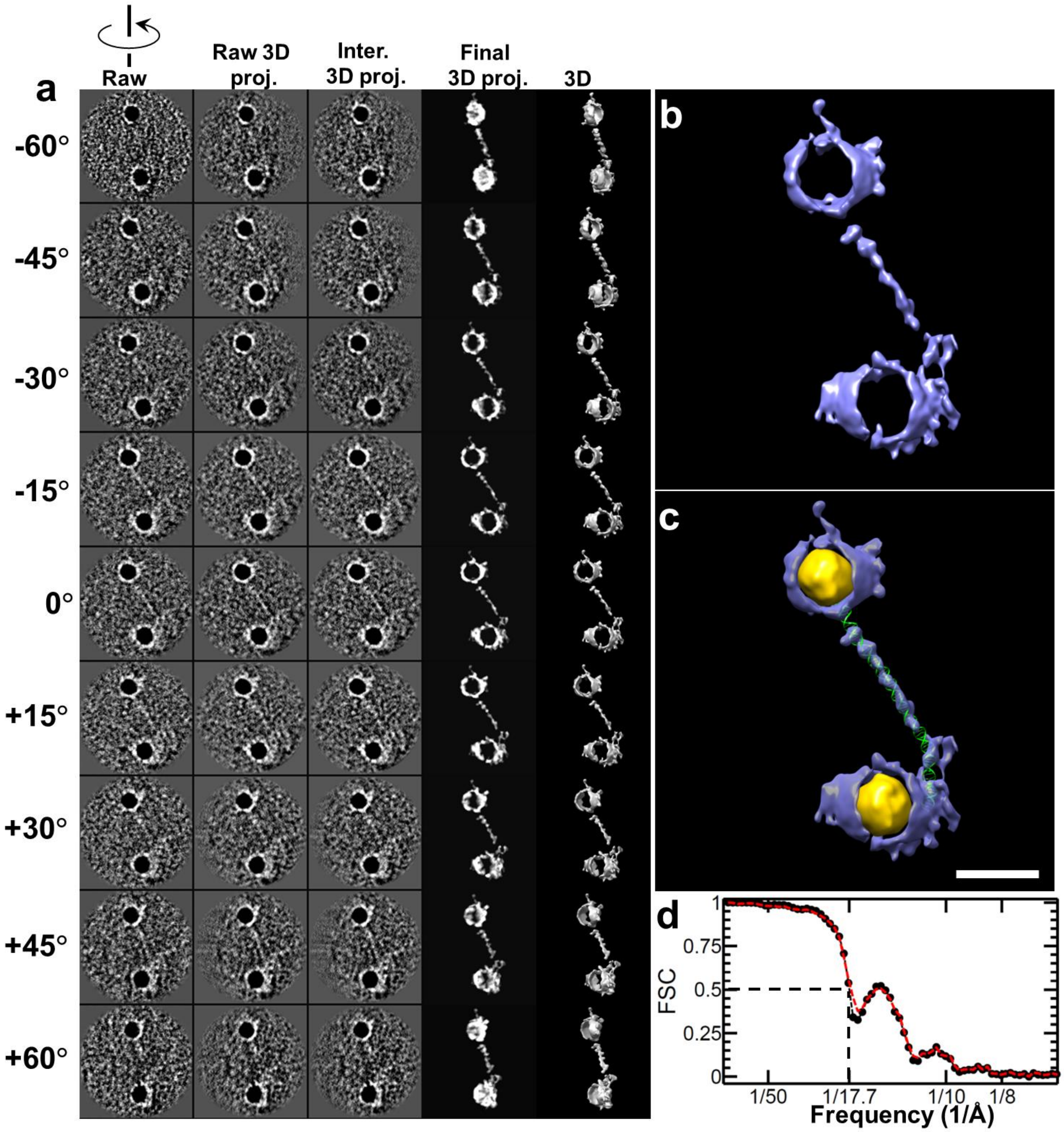

Supplementary Figure 2 | Detailed IPET reconstruction processes on the third DNA-nanogold conjugate. **(a)** Nine representative tilt views (first column, SNR of DNA portion: ~0.51), their corresponding projections on the intermediate 3D reconstructions from major iterations (second to fourth columns) and the final 3D density map (fifth column) are shown. **(b)** The final 3D density map (SNR of the DNA portion: ~3.13) and **(c)** its overlaid 3D density map (final map in gray and its reversed map in gold) display the overall conformation of the DNA-nanogold conjugate. A new conformation of dsDNA was obtained (green ribbon) by flexibly docking the dsDNA standard model into the fabric density between the nanogold particles using the targeted molecular dynamics (TMD) simulations. **(d)** The Fourier shell correlation (FSC) curves under including (black line) and excluding (red line) nanogold portions showed that the resolution of the final 3D reconstruction was ~17.7 Å. Scale bar, 10 nm.

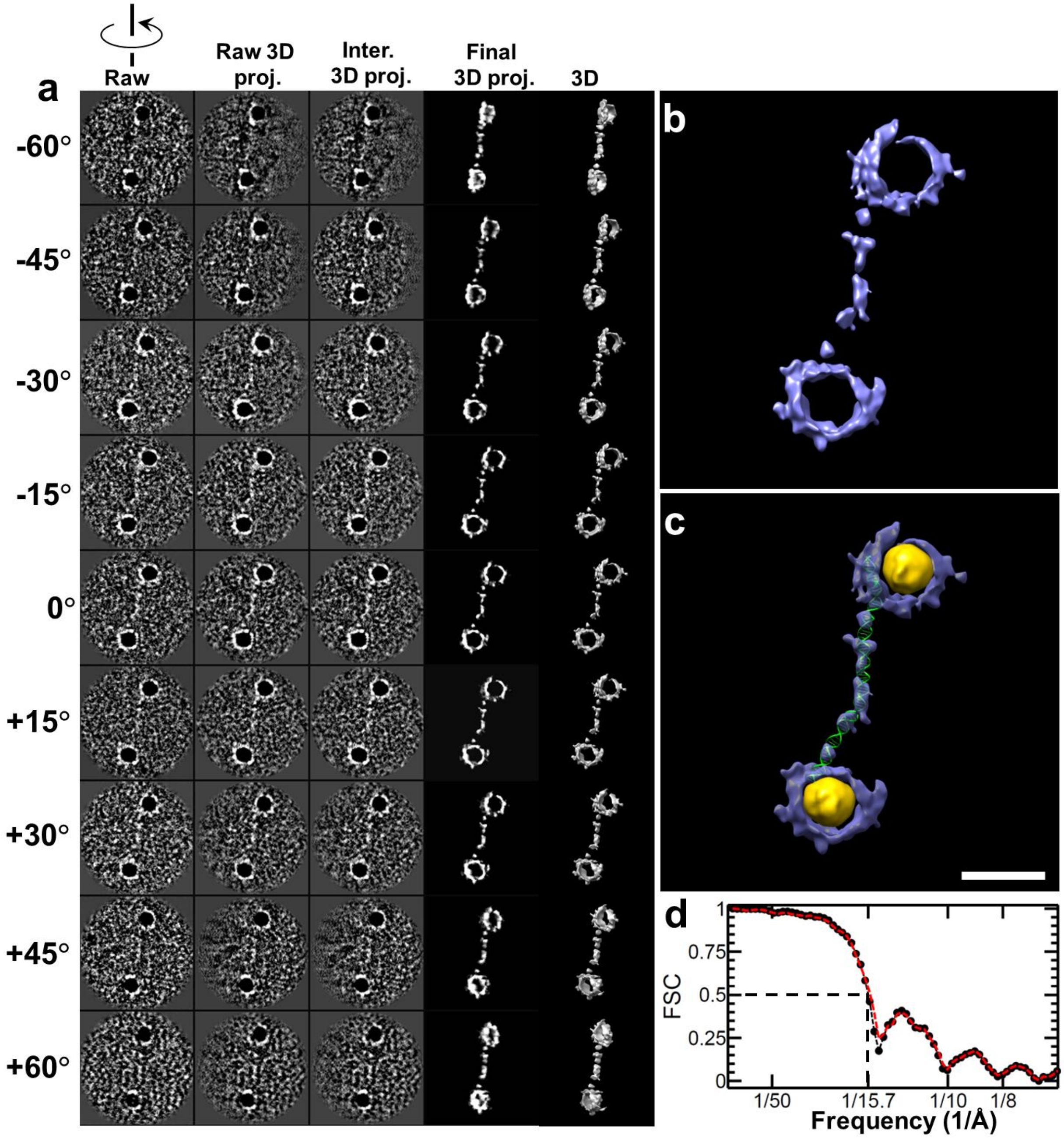

Supplementary Figure 3 | Detailed IPET reconstruction processes on the fourth DNA-nanogold conjugate. **(a)** Nine representative tilt views (first column, SNR of DNA portion: ~0.41), their corresponding projections on the intermediate 3D reconstructions from major iterations (second to fourth columns) and the final 3D density map (fifth column) of the third particle of the DNA-nanogold conjugate are shown. **(b)** The final 3D density map (SNR of DNA portion: ~3.24) and **(c)** its overlaid 3D density map (final map in gray and its reversed map in gold) display the overall conformation of the DNA-nanogold conjugate. A new conformation of dsDNA was obtained (green ribbon) by flexibly docking the dsDNA standard model into the fabric density between the nanogold particles using TMD simulations. **(d)** The FSC curves under including (black line) and excluding (red line) nanogold portions showed that the resolution of the final 3D reconstruction was ~15.7 Å. Scale bar, 10 nm.

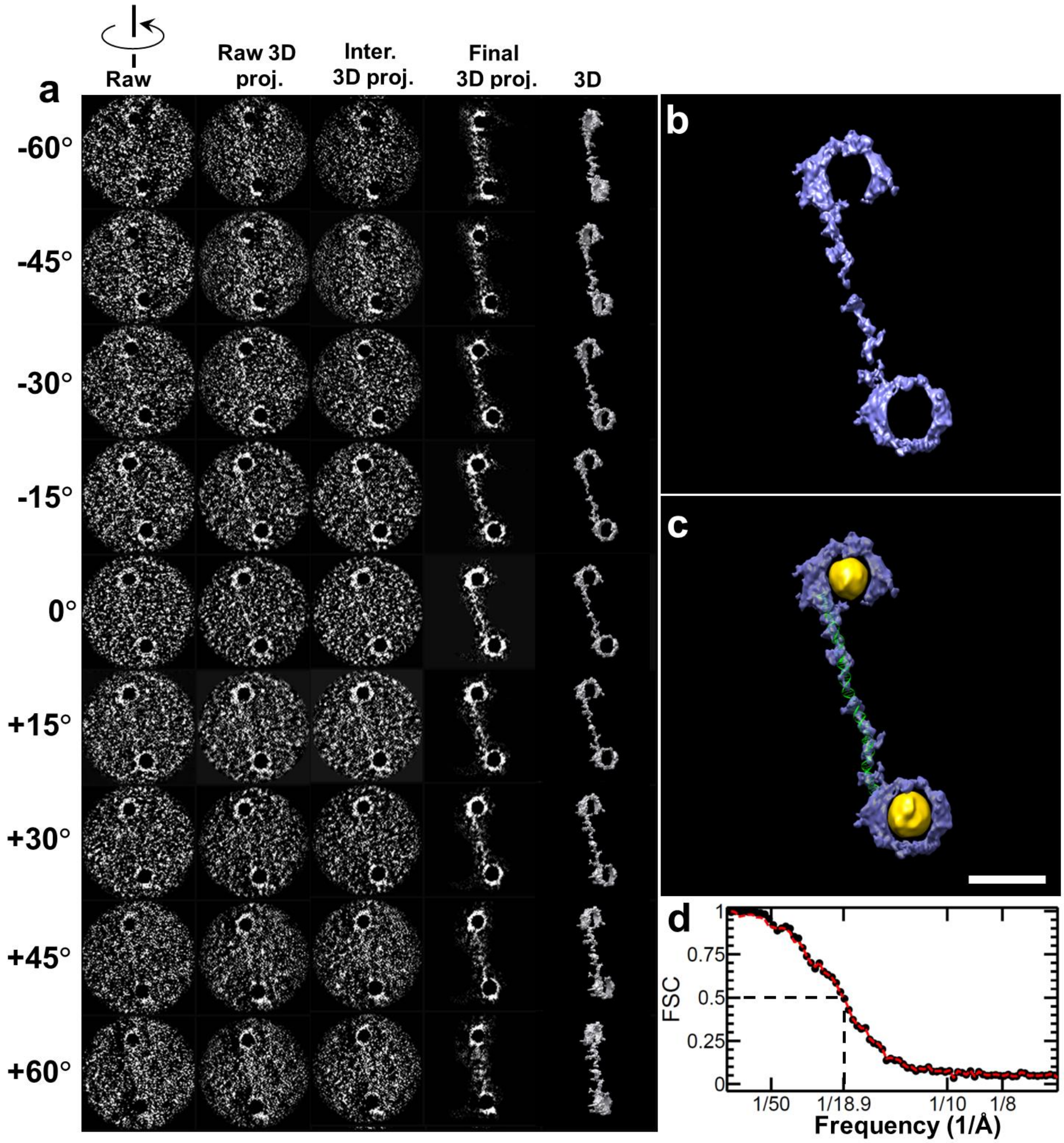

Supplementary Figure 4 | Detailed IPET reconstruction processes on the fifth DNA-nanogold conjugate. **(a)** Nine representative tilt views (first column, SNR of DNA portion: ~0.10), their corresponding projections on the intermediate 3D reconstructions from major iterations (second to fourth columns) and the final 3D density map (fifth column) of the third particle of the DNA-nanogold conjugate are shown. **(b)** The final 3D density map (SNR of DNA portion: ~1.36) and **(c)** its overlaid 3D density map (final map in gray and its reversed map in gold) display the overall conformation of the DNA-nanogold conjugate. A new conformation of dsDNA was obtained (green ribbon) by flexibly docking the dsDNA standard model into the fabric density between the nanogold particles using TMD simulations. **(d)** The FSC curves under including (black line) and excluding (red line) nanogold portions showed that the resolution of the final 3D reconstruction was ~18.9 Å. Scale bar, 10 nm.

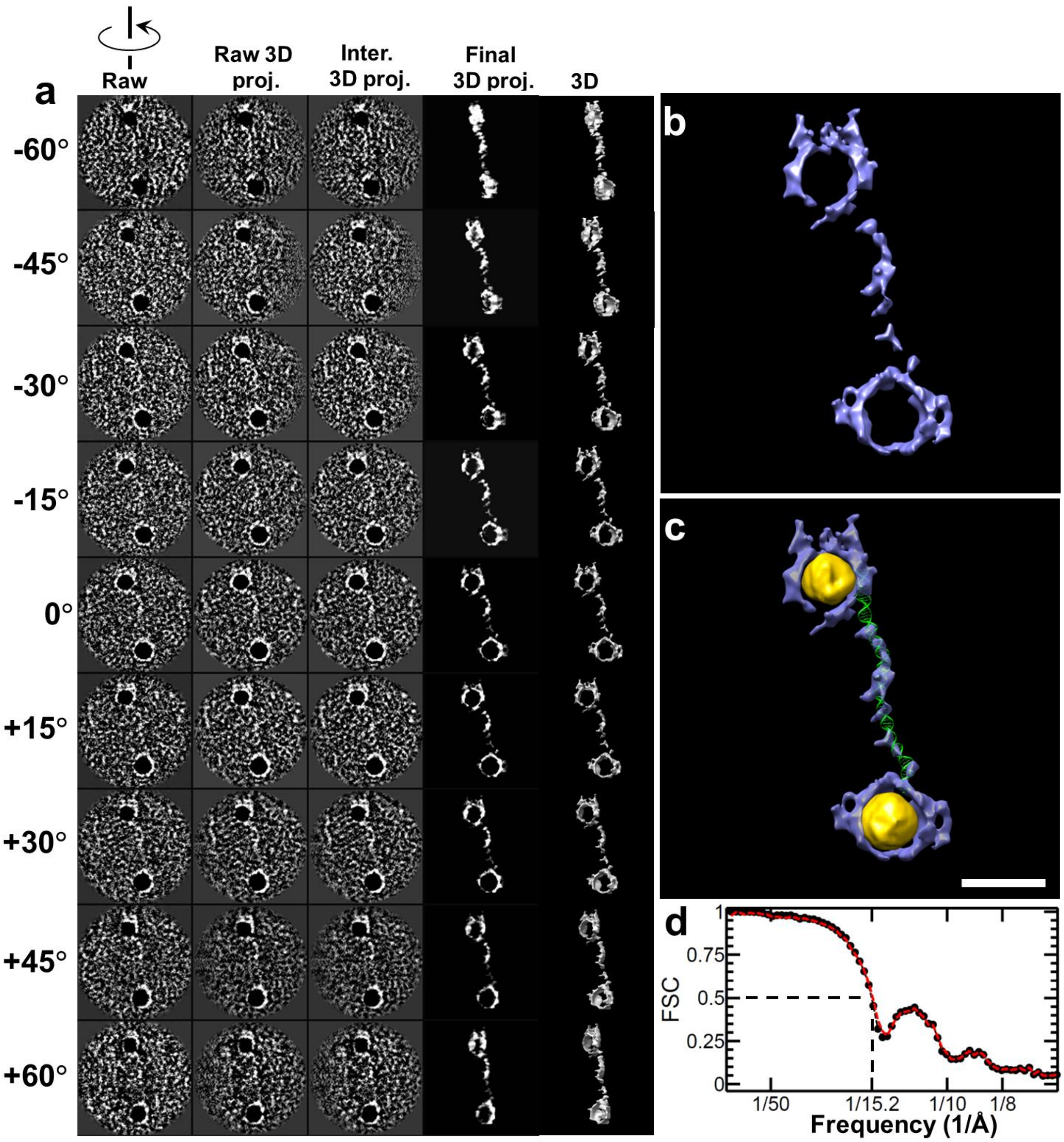

Supplementary Figure 5 | Detailed IPET reconstruction processes on the sixth DNA-nanogold conjugate. **(a)** Nine representative tilt views (first column, SNR of DNA portion: ~0.30), their corresponding projections on the intermediate 3D reconstructions from major iterations (second to fourth columns) and the final 3D density map (fifth column) of the third particle of the DNA-nanogold conjugate are shown. **(b)** The final 3D density map (SNR of DNA portion: ~2.78), and **(c)** its overlaid 3D density map (final map in gray and its reversed map in gold) display the overall conformation of the DNA-nanogold conjugate. A new conformation of dsDNA was obtained (green ribbon) by flexibly docking the dsDNA standard model into the fabric density between the nanogold particles using TMD simulations. **(d)** The FSC curves under including (black line) and excluding (red line) nanogold portions showed that the resolution of the final 3D reconstruction was ~15.2 Å. Scale bar, 10 nm.

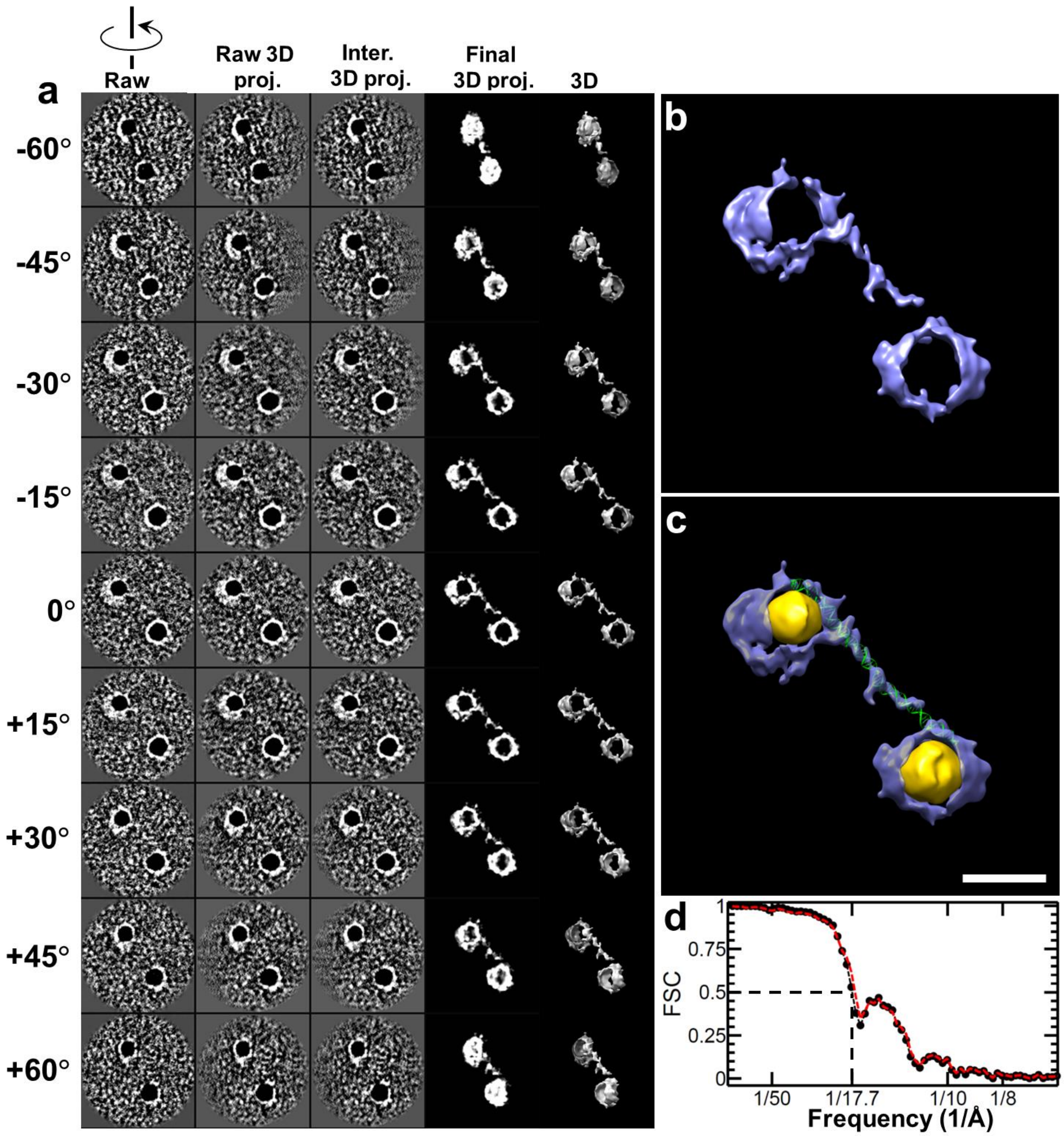

Supplementary Figure 6 | Detailed IPET reconstruction processes on the seventh DNA-nanogold conjugate. **(a)** Nine representative tilt views (first column, SNR of DNA portion: ~0.31), their corresponding projections on the intermediate 3D reconstructions from major iterations (second to fourth columns) and the final 3D density map (fifth column) of the third particle of the DNA-nanogold conjugate are shown. **(b)** The final 3D density map (SNR of DNA portion: ~3.11) and **(c)** its overlaid 3D density map (final map in gray and its reversed map in gold) display the overall conformation of the DNA-nanogold conjugate. A new conformation of dsDNA was obtained (green ribbon) by flexibly docking the dsDNA standard model into the fabric density between the nanogold particles using TMD simulations. **(d)** The FSC curves under including (black line) and excluding (red line) nanogold portions showed that the resolution of the final 3D reconstruction was ~17.7 Å. Scale bar, 10 nm.

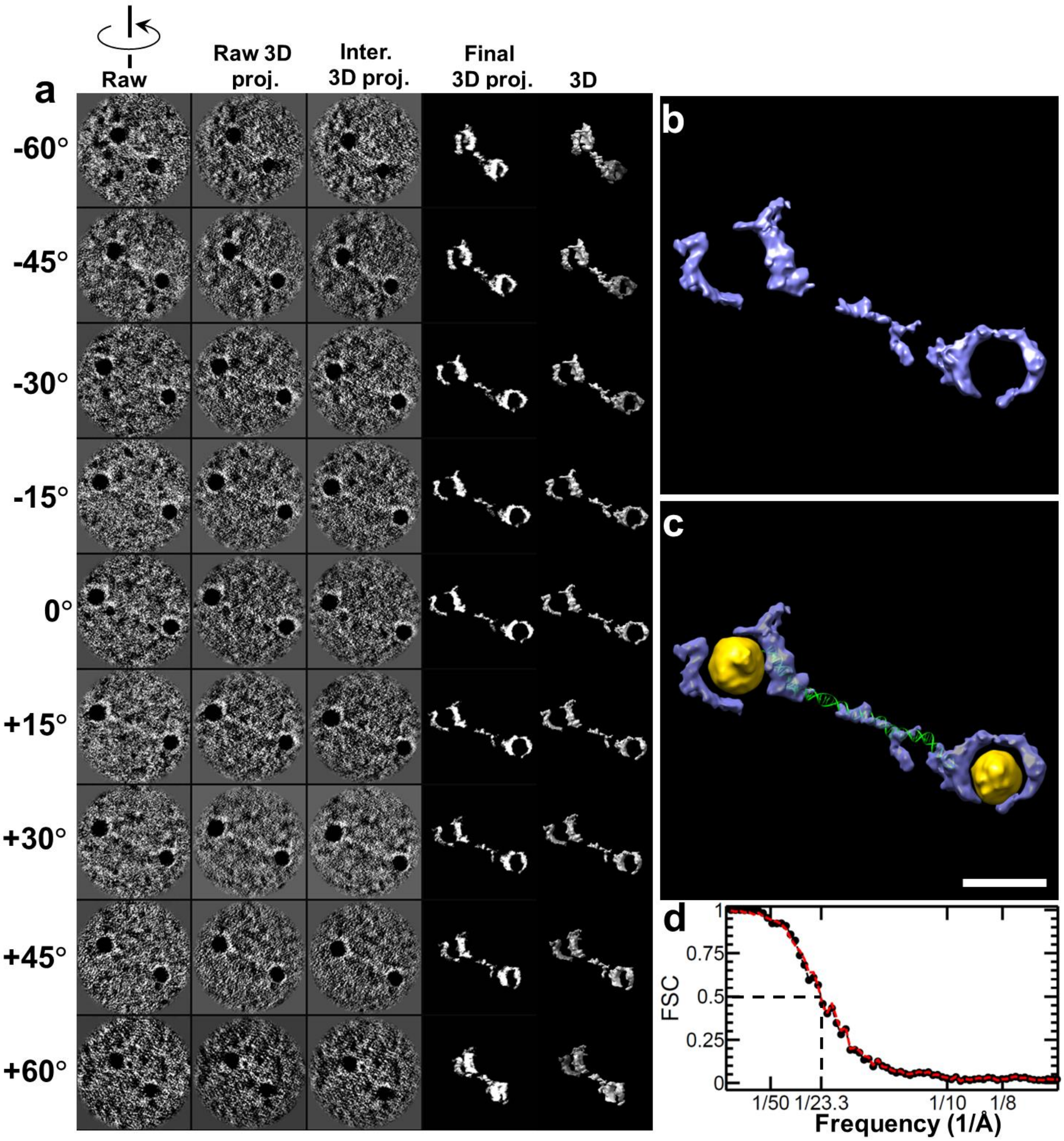

Supplementary Figure 7 | Detailed IPET reconstruction processes on the eighth DNA-nanogold conjugate. **(a)** Nine representative tilt views (first column, SNR of DNA portion: ~0.23), their corresponding projections on the intermediate 3D reconstructions from major iterations (second to fourth columns) and the final 3D density map (fifth column) of the third particle of the DNA-nanogold conjugate are shown. **(b)** The final 3D density map (SNR of DNA portion: ~1.58), and **(c)** its overlaid 3D density map (final map in gray and its reversed map in gold) display the overall conformation of the DNA-nanogold conjugate. A new conformation of dsDNA was obtained (green ribbon) by flexibly docking the dsDNA standard model into the fabric density between the nanogold particles using TMD simulations. **(d)** The FSC curves under including (black line) and excluding (red line) nanogold portions showed that the resolution of the final 3D reconstruction was ~23.3 Å. Scale bar, 10 nm.

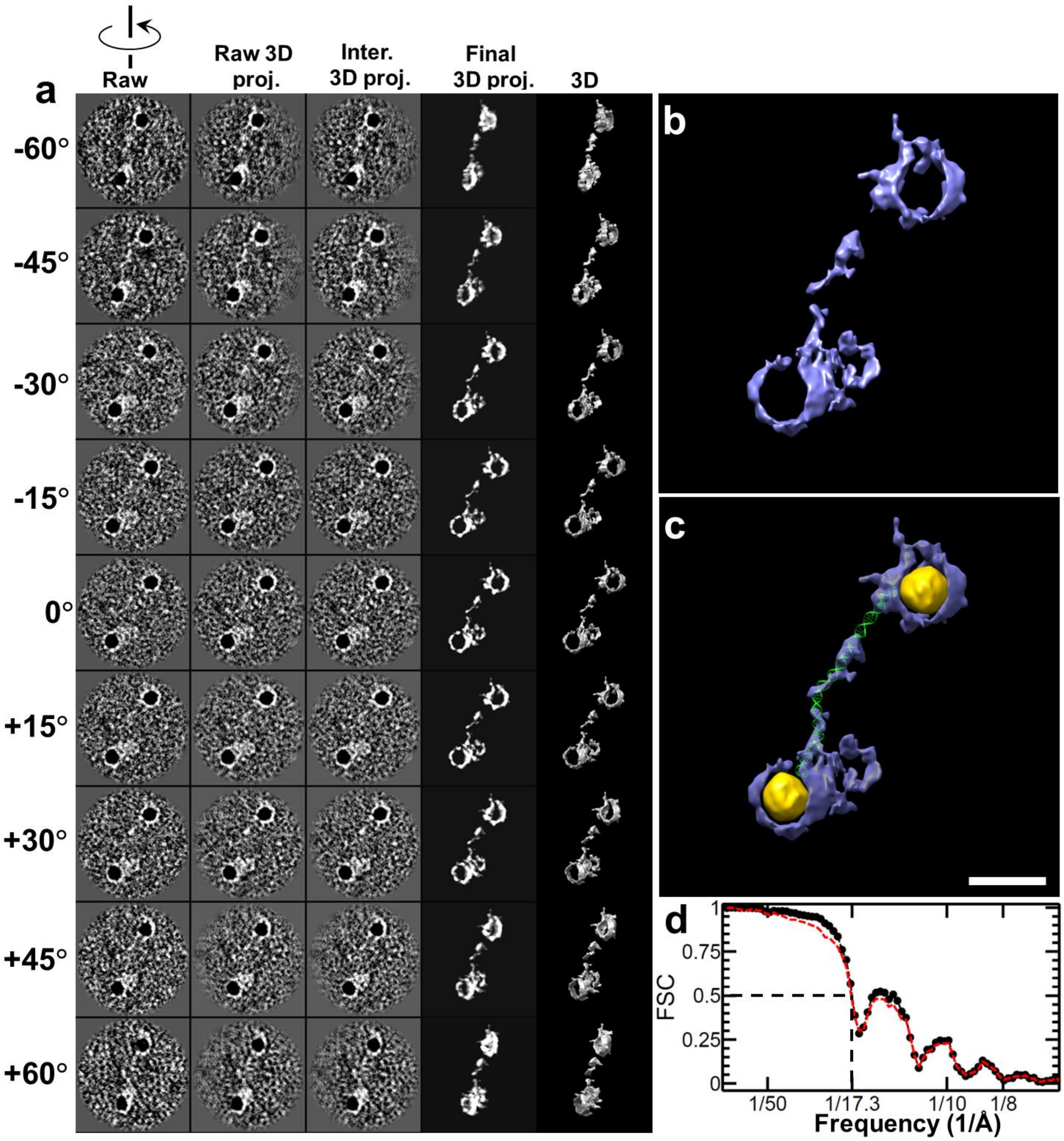

Supplementary Figure 8 | Detailed IPET reconstruction processes on the ninth DNA-nanogold conjugate. **(a)** Nine representative tilt views (first column, SNR of DNA portion: ~0.56), their corresponding projections on the intermediate 3D reconstructions from major iterations (second to fourth columns) and the final 3D density map (fifth column) of the third particle of the DNA-nanogold conjugate are shown. **(b)** The final 3D density map (SNR of DNA portion: ~3.18), and **(c)** its overlaid 3D density map (final map in gray and its reversed map in gold) display the overall conformation of the DNA-nanogold conjugate. A new conformation of dsDNA was obtained (green ribbon) by flexibly docking the dsDNA standard model into the fabric density between the nanogold particles using TMD simulations. **(d)** The FSC curves under including (black line) and excluding (red line) nanogold portions showed that the resolution of the final 3D reconstruction was ~17.3 Å. Scale bar, 10 nm.

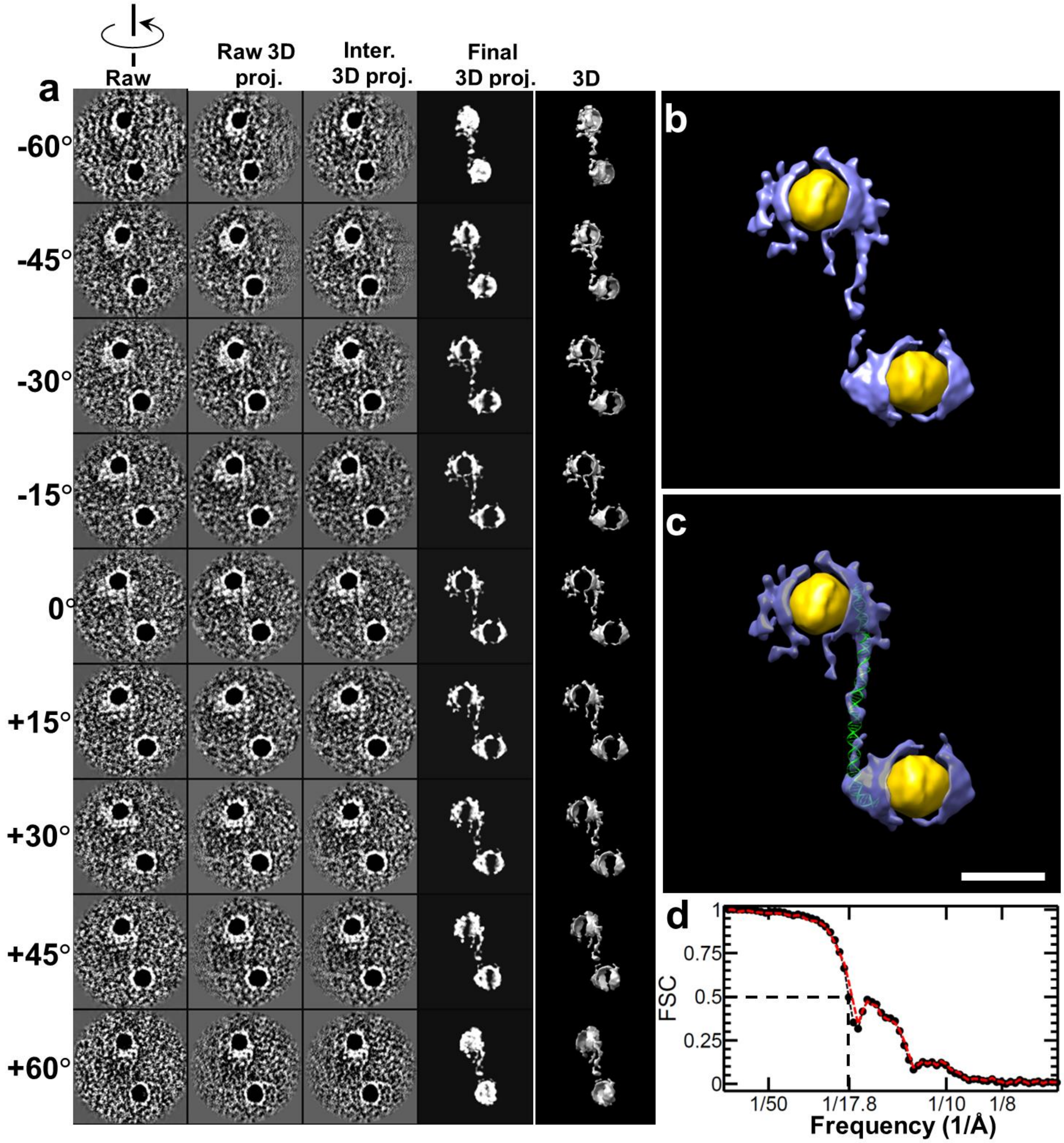

Supplementary Figure 9 | Detailed IPET reconstruction processes on the tenth DNA-nanogold conjugate. **(a)** Nine representative tilt views (first column, SNR of DNA portion: ~0.55), their corresponding projections on the intermediate 3D reconstructions from major iterations (second to fourth columns) and the final 3D density map (fifth column) of the third particle of the DNA-nanogold conjugate are shown. **(b)** The final 3D density map (SNR of DNA portion: ~3.46) and **(c)** its overlaid 3D density map (final map in gray and its reversed map in gold) display the overall conformation of the DNA-nanogold conjugate. A new conformation of dsDNA was obtained (green ribbon) by flexibly docking the dsDNA standard model into the fabric density between the nanogold particles using TMD simulations. **(d)** The FSC curves under including (black line) and excluding (red line) nanogold portions showed that the resolution of the final 3D reconstruction was ~17.8 Å. Scale bar, 10 nm.

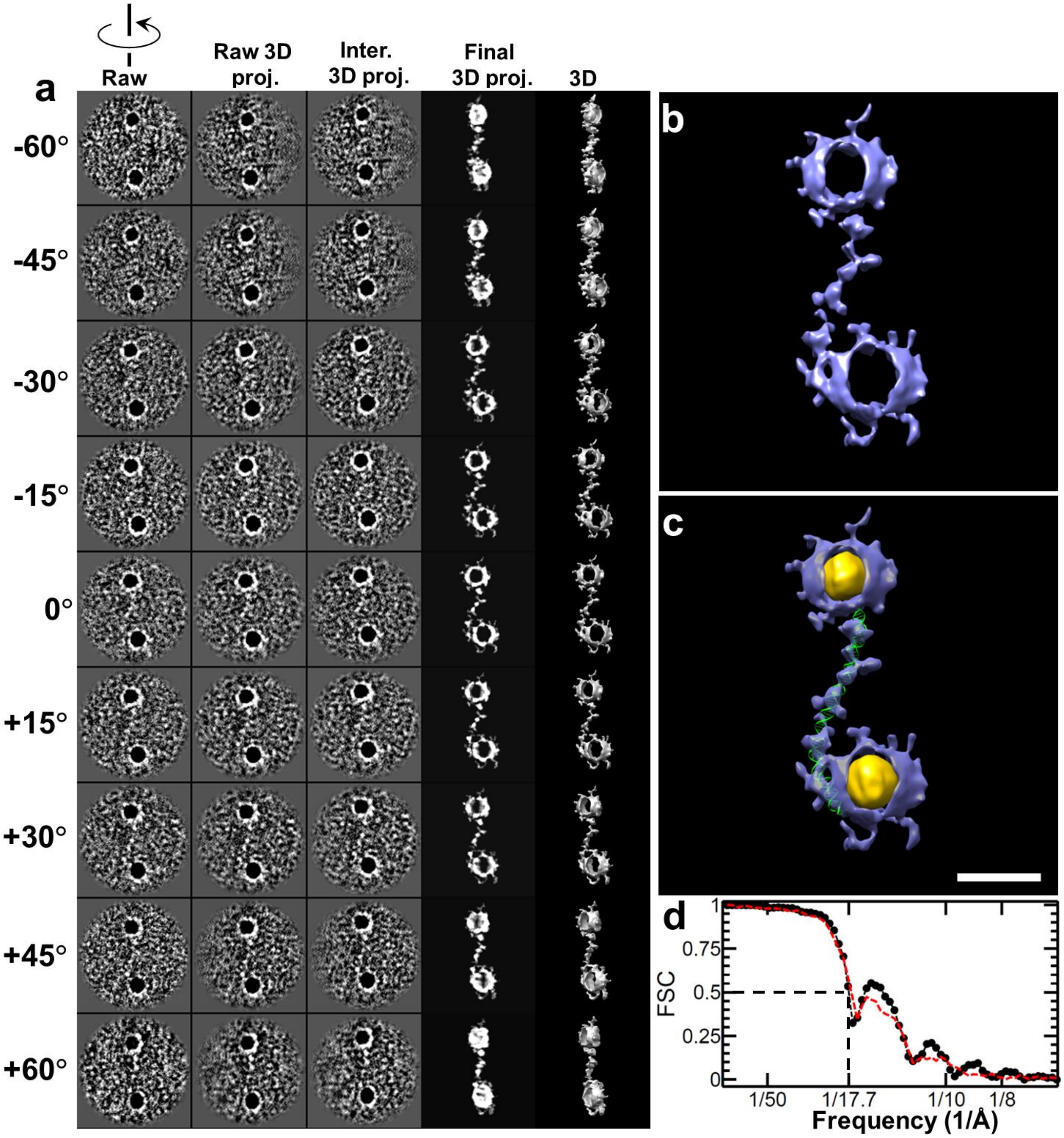

Supplementary Figure 10 | Detailed IPET reconstruction processes on the eleventh DNA-nanogold conjugate. **(a)** Nine representative tilt views (first column, SNR of DNA portion: ~0.23), their corresponding projections on the intermediate 3D reconstructions from major iterations (second to fourth columns) and the final 3D density map (fifth column) of the third particle of the DNA-nanogold conjugate are shown. **(b)** The final 3D density map (SNR of DNA portion: ~2.90) and **(c)** its overlaid 3D density map (final map in gray and its reversed map in gold) display the overall conformation of the DNA-nanogold conjugate. A new conformation of dsDNA was obtained (green ribbon) by flexibly docking the dsDNA standard model into the fabric density between the nanogold particles using TMD simulations. **(d)** The FSC curves under including (black line) and excluding (red line) nanogold portions showed that the resolution of the final 3D reconstruction was ~17.7 Å. Scale bar, 10 nm.

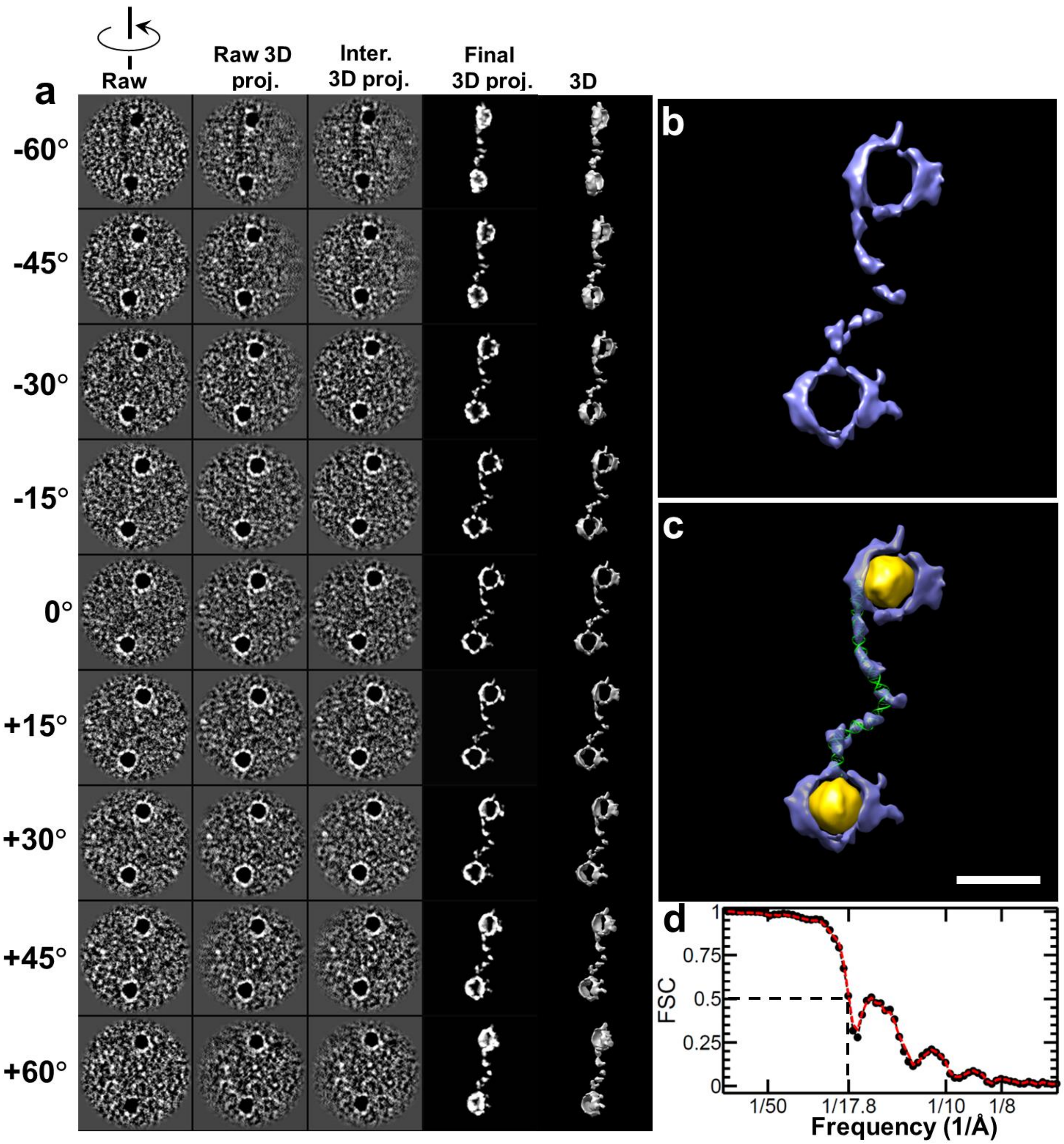

Supplementary Figure 11 | Detailed IPET reconstruction processes on the twelfth DNA-nanogold conjugate. **(a)** Nine representative tilt views (first column, SNR of DNA portion: ~0.32), their corresponding projections on the intermediate 3D reconstructions from major iterations (second to fourth columns) and the final 3D density map (fifth column) of the third particle of the DNA-nanogold conjugate are shown. **(b)** The final 3D density map (SNR of DNA portion: ~3.34) and **(c)** its overlaid 3D density map (final map in gray and its reversed map in gold) display the overall conformation of the DNA-nanogold conjugate. A new conformation of dsDNA was obtained (green ribbon) by flexibly docking the dsDNA standard model into the fabric density between the nanogold particles using TMD simulations. **(d)** The FSC curves under including (black line) and excluding (red line) nanogold portions showed that the resolution of the final 3D reconstruction was ~17.8 Å. Scale bar, 10 nm.

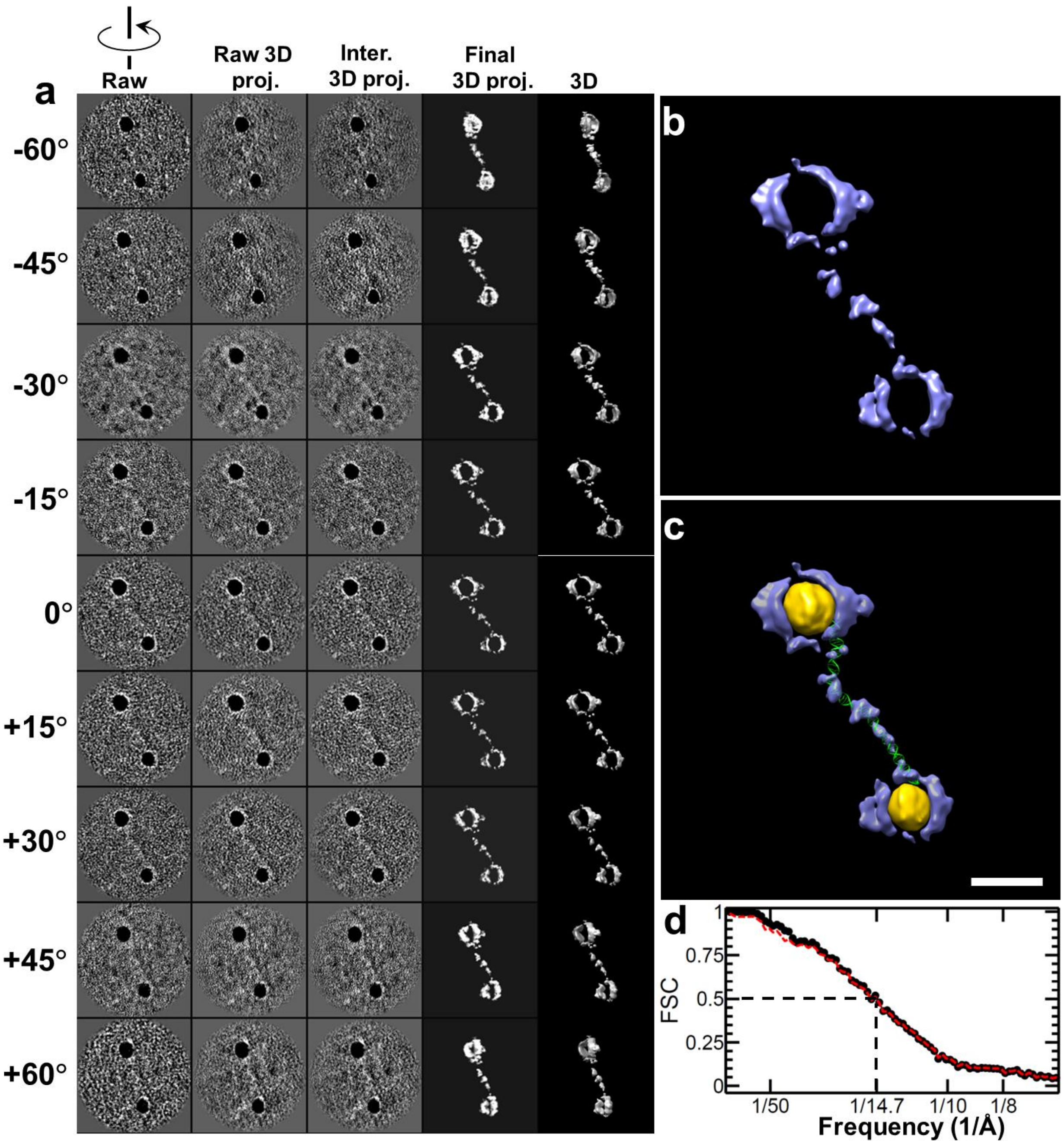

Supplementary Figure 12 | Detailed IPET reconstruction processes on the thirteenth DNA-nanogold conjugate. **(a)** Nine representative tilt views (first column, SNR of DNA portion: ~0.28), their corresponding projections on the intermediate 3D reconstructions from major iterations (second to fourth columns) and the final 3D density map (fifth column) of the third particle of the DNA-nanogold conjugate are shown. **(b)** The final 3D density map (SNR of DNA portion: ~1.93) and **(c)** its overlaid 3D density map (final map in gray and its reversed map in gold) display the overall conformation of the DNA-nanogold conjugate. A new conformation of dsDNA was obtained (green ribbon) by flexibly docking the dsDNA standard model into the fabric density between the nanogold particles using TMD simulations. **(d)** The FSC curves under including (black line) and excluding (red line) nanogold portions showed that the resolution of the final 3D reconstruction was ~14.7 Å. Scale bar, 10 nm.

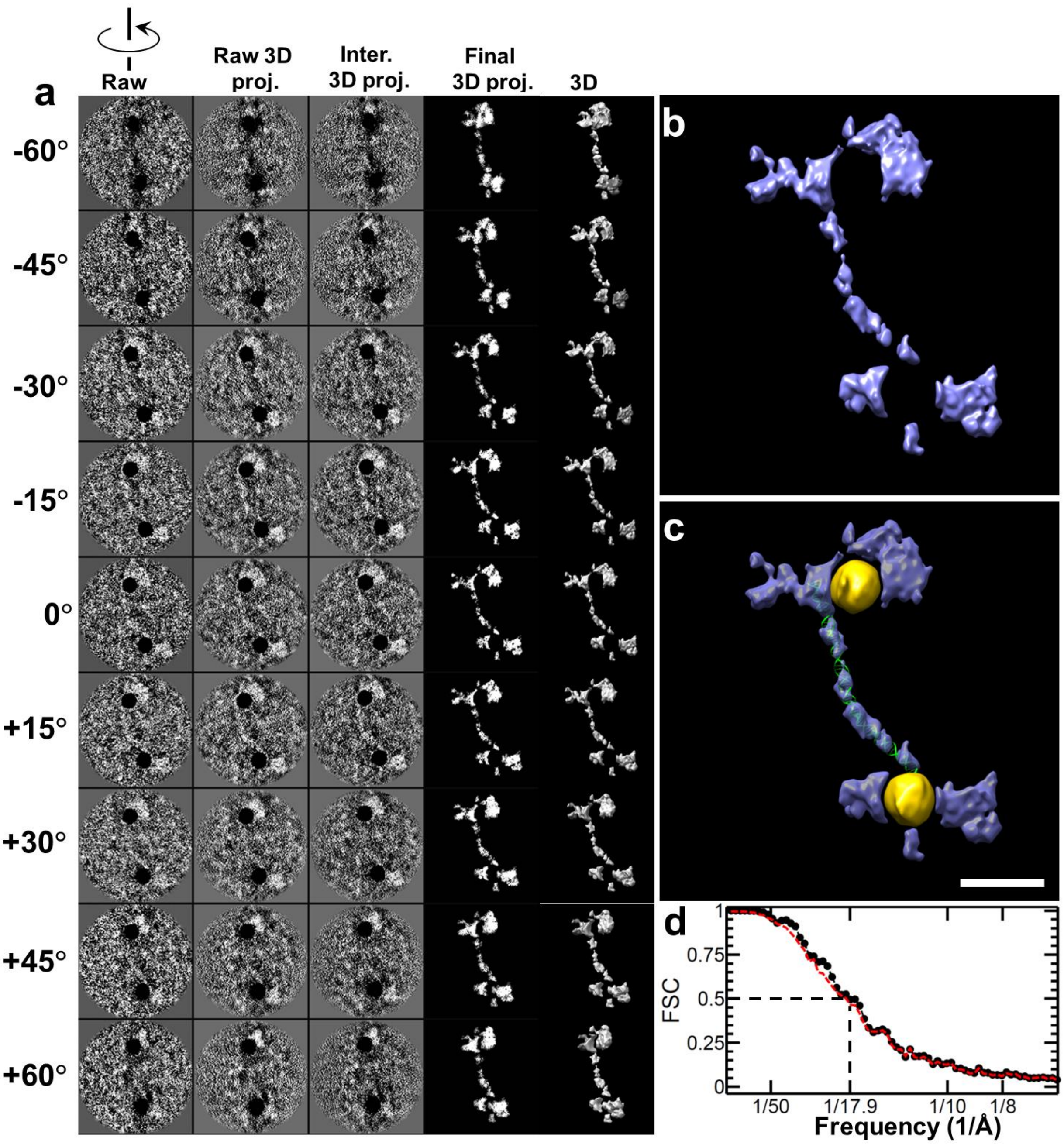

Supplementary Figure 13 | Detailed IPET reconstruction processes on the fourteenth DNA-nanogold conjugate. **(a)** Nine representative tilt views (first column, SNR of DNA portion: ~0.22), their corresponding projections on the intermediate 3D reconstructions from major iterations (second to fourth columns) and the final 3D density map (fifth column) of the third particle of the DNA-nanogold conjugate are shown. **(b)** The final 3D density map (SNR of DNA portion: ~1.45) and **(c)** its overlaid 3D density map (final map in gray and its reversed map in gold) display the overall conformation of the DNA-nanogold conjugate. A new conformation of dsDNA was obtained (green ribbon) by flexibly docking the dsDNA standard model into the fabric density between the nanogold particles using TMD simulations. **(d)** The curves under including (black line) and excluding (red line) nanogold portions showed that the resolution of the final 3D reconstruction was ~17.9 Å. Scale bar, 10 nm.

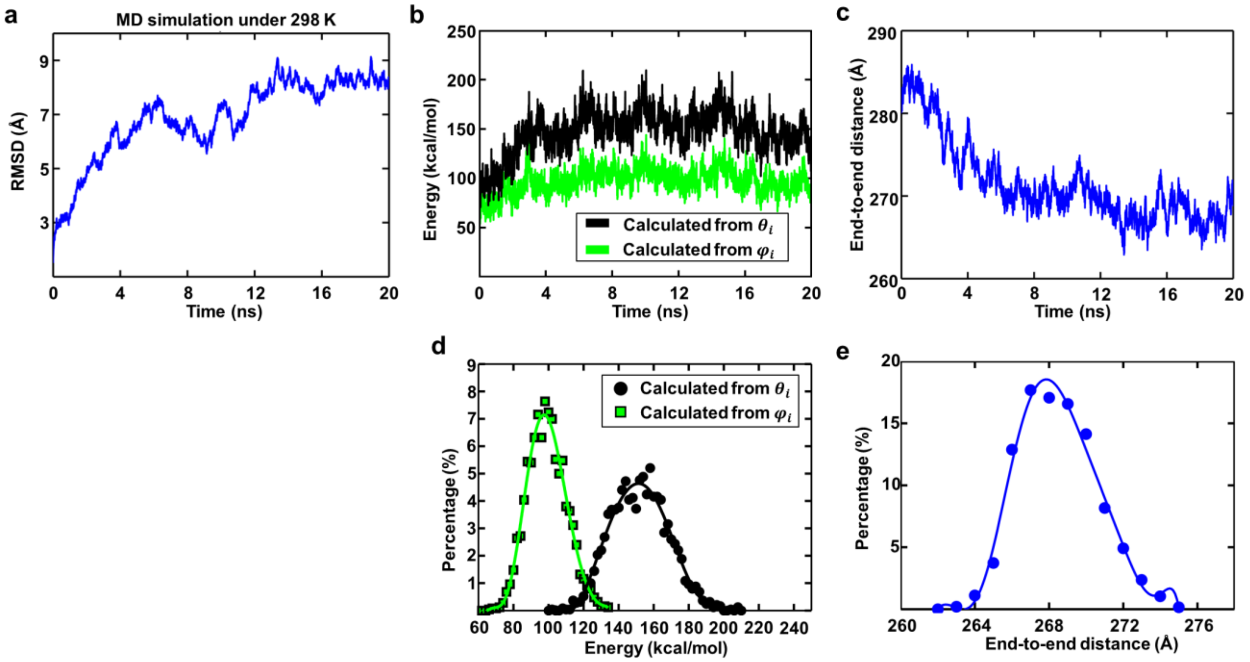

Supplementary Figure 14 | Statistical analyses of the bending energies and lengths of an 84-base-pair dsDNA in solution at 298 K via MD simulations. The equilibration of dsDNA at 298 K, 1 atm and a 0.15 M salt concentration was monitored using **(a)** the RMSD, **(b)** the DNA bending energy and **(c)** the end-to-end distance of DNA. After 8 ns of simulation, the bending energy converged to 99.1 ± 10.9 Kcal/mol (calculated from φ_i) or 152.1 ± 16.1 kcal/mol (calculated from θ_i), and the end-to-end distance converged to 268.5 ± 2.1 Å (averaged over the last 10 ns). **(d)** Histogram of the bending energy in the last 10 ns of simulation. For the statistical analysis, the histograms were normalized and fit using a ninth-degree polynomial function. The peak populations occurred at ~97.4 ± 1.0 kcal/mol (~7.1%, calculated from φ_i) and ~151.3 ± 1.0 kcal/mol (~4.6%, calculated from θ_i). A sampling step of 2 kcal/mol was used for the histograms. **(e)** Histogram of the end-to-end distance in the last 10 ns of the simulation. For the statistical analysis, the histograms were normalized and fit using a ninth-degree polynomial function. The peak populations occurred at ~267.9 ± 0.5 Å (~18.5%). A sampling step of 1 Å was used for the histograms.

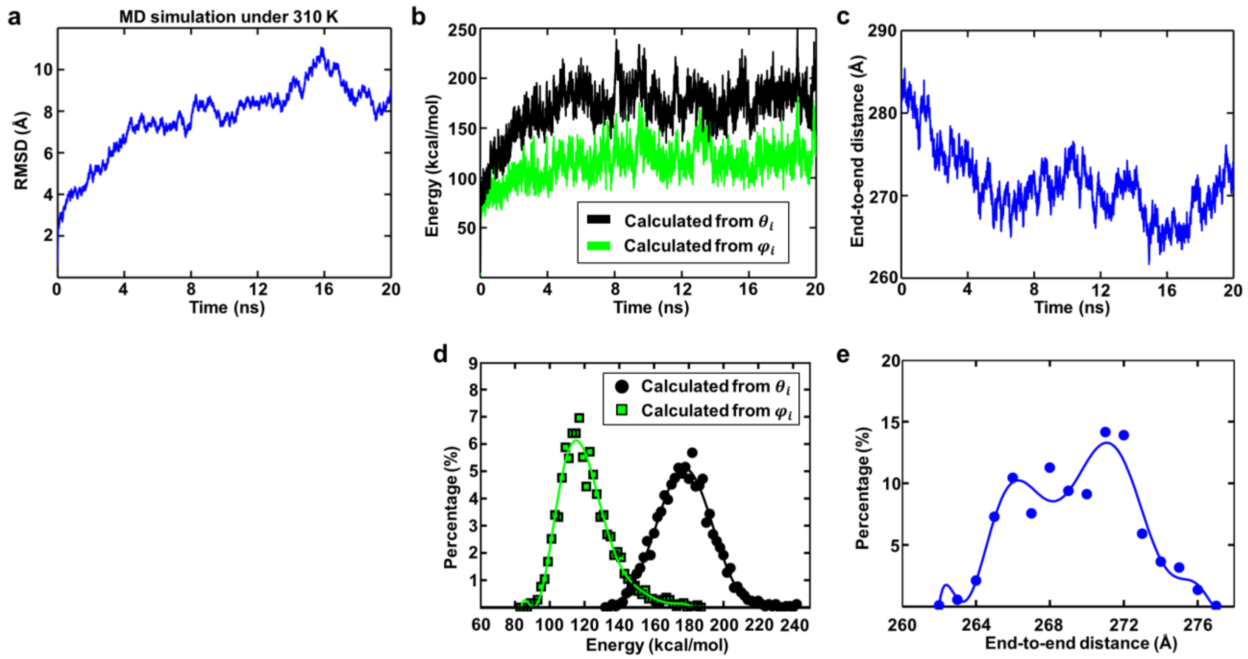

Supplementary Figure 15 | Statistical analyses of the bending energies and lengths of an 84-base-pair dsDNA in solution at 310 K via MD simulations. The equilibration of the dsDNA in physiological solution (310 K, 1 atm, 0.15 M salt concentration) was monitored using **(a)** the root mean-square deviation (RMSD), **(b)** the DNA bending energy, and **(c)** the end-to-end distance of DNA. After 8 ns of simulation, the bending energy converged to 120.8 ± 14.7 kcal/mol (calculated from φ_i) or 177.9 ± 15.6 kcal/mol (calculated from $\theta_{i\ i}$), whereas the end-to-end distance converged to 269.5 ± 2.9 Å (averaged from the last 10 ns). **(d)** Histogram of the bending energy during the last 10 ns of the simulation. For the statistical analysis, the histograms were normalized and fit using a ninth-degree polynomial function. The peak populations occurred at ~115.1 ± 1.0 kcal/mol (~6.1%, calculated from φ_i) and ~177.5 ± 1.0 kcal/mol (~5.1%, calculated from θ_i). The histograms have a sampling step of 2 kcal/mol. **(e)** Histogram of the end-to-end distance in the last 10 ns of the simulation. For the statistical analysis, the histograms were normalized and fit using a ninth-degree polynomial function. The peak populations occurred at ~271.1 ± 0.5 Å (~13.3%). A sampling step of 1 Å was used for the histograms.

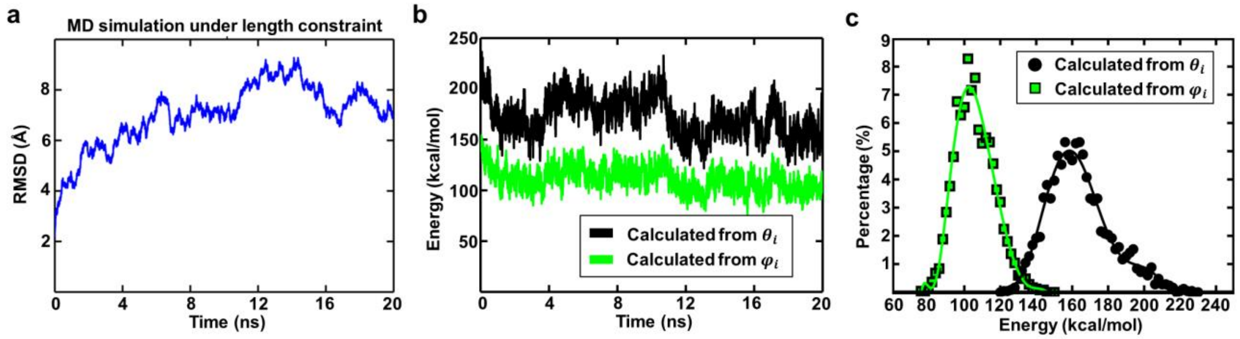

Supplementary Figure 16 | Statistical analyses of the bending energies of an 84-base-pair dsDNA with a constrained length of 241.0 Å in solution via MD simulations. **(a)** The equilibration of dsDNA with a length of 241.0 Å at room temperature (293 K), 1 atm and a 0.15 M salt concentration was monitored using the RMSD. **(b)** The DNA bending energy converged to 105.8 ± 10.7 kcal/mol (calculated from φ_i) or 163.6 ± 17.4 kcal/mol (calculated from θ_i) after 8 ns of simulation. **(c)** Histogram of the bending energy during the last 10 ns of simulation. For the statistical analysis, the histograms were normalized and fit using a ninth-degree polynomial function. The peak populations occurred at ~102.7 ± 1.0 kcal/mol (~7.3%, calculated from φ_i) and ~158.4 ± 1.0 kcal/mol (~5.0%, calculated from θ_i). A sampling step of 2 kcal/mol was used in the histograms.

Data information of all 14 IPET 3D EM density maps

Fig. ID	FSC Resolution (Å)	Center-center Distance (Å)	1st nanogold geometric diameter (Å)	2nd nanogold geometric diameter (Å)	Average width of DNA portion (Å)	SNR (measured from 2Ds)	SNR (measured from 3D)	EMDB ID	Display contour level
**2B**	14.7	313.4	73.3	71.9	18.3±3.8	0.31	2.44	2948	0.251
**2F**	17.1	261.5	71.4	65.1	20.3±3.3	0.56	3.26	2949	0.299
**S2**	17.7	285.6	59.4	66.5	21.1±3.2	0.51	3.13	2950	0.310
**S3**	15.7	297.3	63.9	61.6	20.8±3.9	0.41	3.24	2951	0.309
**S4**	18.9	329.3	63.6	58.7	24.4±6.4	0.10	1.36	2952	0.171
**S5**	15.2	302.6	63.5	71.6	18.4±3.3	0.30	2.78	2953	0.285
**S6**	17.7	250.3	64.2	70.6	20.5±3.3	0.31	3.11	2954	0.280
**S7**	23.3	342.4	68.1	63.0	18.8±3.0	0.23	1.58	2955	0.117
**S8**	17.3	328.6	60.4	59.5	21.6±7.2	0.56	3.18	2956	0.298
**S9**	17.8	249.9	68.4	74.7	17.2±3.3	0.55	3.46	2957	0.357
**S10**	17.7	253.2	55.3	62.2	21.9±3.7	0.23	2.90	2958	0.221
**S11**	17.8	278.9	64.1	64.5	19.8±4.1	0.32	3.34	2959	0.305
**S12**	14.7	315.6	73.7	67.6	20.4±5.5	0.28	1.93	2960	0.129
**S13**	17.9	266.2	68.6	65.3	24.0±5.0	0.22	1.45	2961	0.061
**Average**	-	291.1±31.9	65.6±5.3	65.9±4.9	20.5±4.4	-	-	-	-

Supplementary Table 1 | Parameters of the 14 reconstructed DNA-nanogold conjugates.

Length and bending energy analyses on an 84bp dsDNA

(based on WLC model, Persistent length 50nm)

	Length Analyses				Bending Energy Analyses			
	2D projection length (Å)		3D length (Å)		Bending energy based on angle definition of φ (kcal/mol)		Bending energy based on angle definition of θ (kcal/mol)	
	Mean value	Peak population	Mean value	Peak population	Based on all 84bp	Based on central 42bp	Based on all 84bp	Based on central 42bp
**Our EM measurements**								
Cryo-EM 2D images	255.3 ± 48.7	286.4	-	-	-	-	-	-
NS 2D images	245.5 ± 62.6	287.0	-	-	-	-	-	-
IPET 3D density maps, and Manually fitted DNA model + MDFF + MD simulations)	-	-	291.1 ± 31.9[a]	-	115.6±22.8	102.6±13.4	168.9±38.7	147.2±20.3
**Others measurements**								
SAXS (ref: 25)	-	-	~280[a]	~320[a]	-	-	-	-
In-situ TEM (ref: 24)	~180[a]	-	-	-	-	-	-	-
WLC model prediction (ref: 25)	-	-	~300[a]	~320[a]	-	-	-	-
**MD simulation prediction**								
Smoothly fitted DNA model into EM map	-	-	-	-	8.5±4.1	8.3±2.7	11.0±3.3	11.0±2.1
Smoothly fitted model + Energy minimization	-	-	-	-	35.2±5.5	35.4±4.2	51.6±9.7	47.7±5.3
Smoothly fitted model + Energy minimization + 0.1ns MD simulations	-	-	-	-	101.0±11.8	97.2±8.0	132.2±19.6	122.9±11.1
Smoothly fitted model + MDFF	-	-	-	-	124.5±12.3	104.6±10.7	182.2±19.0	150.7±15.6
A straight DNA model + MD simulations at 298K	-	-	268.5 ± 2.1	267.9 ± 0.5	99.1±10.9	93.4±7.6	152.1±16.1	142.2±10.9
A straight DNA model + MD simulations at 310K	-	-	269.5 ± 2.9	271.1 ± 0.5	120.8±14.7	101.9±7.5	177.9±15.6	158.8±12.3
An IPET DNA model + MD simulations + a fixed length constrain at 293K	-	-	-	-	105.8±10.7	104.1±7.2	163.6±17.4	154.4±12.6

[a] Distance was estimated based on the center-to-center distance of nano-gold particles conjugating with dsDNA, instead of the end-to-end distance of dsDNA (from 14 3D density maps).
[b,c] Bending energy was calculated based on two types of bending angle definitions: [b] φ_i based on orientations/central axes of consecutive base-pairs, and [c] θ_i based on centers of consecutive base-pairs.
- No data can be or has been obtained.

Supplementary Table 2 | Comparison of the results of the 84-base-pair dsDNA. The bending energy and length of the 84-base-pair dsDNA from the IPET reconstruction, SAXS, in situ TEM, WLC model prediction and MD simulations were compared.
